# Free-radicals and advanced chemistries involved in cell membrane organization influence oxygen diffusion and pathology treatment

**DOI:** 10.3934/biophy.2017.2.240

**Published:** 2017-04-06

**Authors:** Richard C Petersen

**Affiliations:** 1 Biomaterials, SDB 539, 1919 7th Avenue South, University of Alabama at Birmingham, Birmingham, AL 35294, USA; 2 Biomedical Research Technologies, 3830 Avenida Del Presidente, M/S 36, San Clemente, CA, 92674, USA

**Keywords:** free radicals, crosslink, mechanomolecular, calcium, membrane fluidity, oxygen, antioxidant, cancer, hydroquinone

## Abstract

A breakthrough has been discovered in pathology chemistry related to increasing molecular structure that can interfere with oxygen diffusion through cell membranes. Free radicals can crosslink unsaturated low-viscosity fatty acid oils by chain-growth polymerization into more viscous liquids and even solids. Free radicals are released by mitochondria in response to intermittent hypoxia that can increase membrane molecular organization to reduce fluidity and oxygen diffusion in a possible continuing vicious cycle toward pathological disease. Alternate computational chemistry demonstrates molecular bond dynamics in free energy for cell membrane physiologic movements. Paired electrons in oxygen and nitrogen atoms require that oxygen bonds rotate and nitrogen bonds invert to seek polar nano-environments and hide from nonpolar nano-environments thus creating fluctuating instability at a nonpolar membrane and polar biologic fluid interface. Subsequent mechanomolecular movements provide free energy to increase diffusion by membrane transport of molecules and oxygen into the cell, cell-membrane signaling/recognition/defense in addition to protein movements for enzyme mixing. In other chemistry calcium bonds to membrane phosphates primarily on the outer plasma cell membrane surface to influence the membrane firing threshold for excitability and better seal out water permeation. Because calcium is an excellent metal conductor and membrane phosphate headgroups form a semiconductor at the biologic fluid interface, excess electrons released by mitochondria may have more broad dissipation potential by safe conduction through calcium atomic-sized circuits on the outer membrane surface. Regarding medical conditions, free radicals are known to produce pathology especially in age-related disease in addition to aging. Because cancer cell membranes develop extreme polymorphism that has been extensively followed in research, accentuated easily-visualized free-radical models are developed. In terms of treatment, use of vitamin nutrient supplements purported to be antioxidants that remove free radicals has not proved worthwhile in clinical trials presumably due to errors with early antioxidant measurements based on inaccurate colorimetry tests. However, newer covalent-bond shrinkage tests now provide accurate measurements for free-radical inhibitor hydroquinone and other molecules toward drug therapy.

## 1. Introduction

The consequences for blockage of molecular oxygen at the molecular, cell membrane and tissue levels has implications that provide a better understanding of pathology in most every major medical condition known to mankind. New advancements in free-radical chemistry that increase the lipid molecular carbon-bond saturation and propagate crosslinking between unsaturated carbon-carbon double (C=C) bonds to increase organization [1] can be applied to the basic fluid-mosaic membrane model (F-MMM) [2,3]. According to the original F-MMM in 1972 globular intrinsic proteins were thought to be embedded in a phospholipid matrix and have translational mobility [2]. Due to protein-protein interactions, membranes with a high density of the globular intrinsic proteins have low fluidity to increase structure of some form [4]. Subsequent transmembrane proteins were discovered that link together with many proteins all the way through the plasma cell membrane from the extracellular matrix through the integral globular membrane proteins to cytoskeletal filament proteins [3] that should structure membrane organization more. Further, proteins aggregate by crosslinking especially through the amino acid tyrosine with free-radical covalent bonding that should play some role to increase membrane organization [5,6]. Also, intrinsic proteins extending outside the membrane may be linked through peripheral proteins by crosslinking antibody proteins [4].

For general consideration, as molecular chains increase crosslink density low-viscosity oil changes toward a more viscous oil and even a rigid solid by limiting chain motions with sol to gel phase transitions [1,7–10]. Further, the modulus (approximately stiffness) increases with molecular chain crosslinking whereas chain scission decreases modulus [11]. Free radicals are unstable species with at least one unpaired electron in a molecular orbital that confers extreme high reactivity as an electrophile to seek additional electrons [1]. Free radicals as reactive oxygen species (ROS) at normal lower homeostatic concentrations are thought to be involved in basic cellular physiology such as antimicrobial oxidative bursts to kill pathogens, regulate autophagy to recycle intracellular molecules or organelles as a form of nutrient biosynthesis and also promote cell signaling as an adaptive mechanism to stress [12–18]. However, at high concentrations free-radicals are damaging to lipids, proteins and DNA [12,13,18–24] and also are found to be involved in most important medical conditions studied [13,21,23,24] that includes aging [20,25–28] and cellular senescence [29,30]. Further, free radicals can result in the alteration of normal membrane fluidity to increase rigidity [26,31–35] most importantly with polyunsaturated fatty acid (PUFA) targets that lower in content as an indication crosslinking occurs [31–35]. Membrane fluidity can also decrease as the fatty acid saturated/unsaturated ratio increases [36,37] where saturation decreases spaces between fatty acid chains and makes the membrane less permeable to water [37]. Conversely, membrane fluidity can increase by reducing the fatty acid chain lengths [36] that can occur during free-radical electrophilic attack with hydrolysis on PUFA C=C bonds [1,38,39] by oxidative cleavage forming aldehydes that can move easier into smaller spaces [1]. Increased fluidity can take place because smaller molecules increase diffusion exponentially while longer molecules reduce movement due to bond entanglements [40].

In terms of complex chemistries, early Biophysics provided decisive informative models on protein movements studied through vibrating fluctuations of single bond rotational energies [41,42]. Such rapid alternating sigma (σ) bond rotations have been extended significantly through new advanced computational chemistry and also nitrogen inversions with lipids, sugars and proteins to offer advanced science on free mechanomolecular energy at the unstable but vibrant cell membrane/biologic fluid interface [43]. Constant alternating molecular single-bond rotational motion and nitrogen inversions then provide fluctuating vibrational movements to best explain membrane transport to increase diffusion of other molecules like oxygen, cell membrane recognition/signaling/defense and also enzyme molecular “mixing” action [43]. Subsequent mechanomolecular movements then give the basic static diffusion-oriented fluid mosaic model of membrane structure more overall dynamics for the living cell. Other important fundamental chemistry regarding structure and electron transfer simplification relative to cellular physiology can be applied for membrane organization with calcium on the outer extracellular layer that forms a wide diverse range of mineral structure and cements [44–48].

## 2. Reactive Secondary Sequence Polymerization and Lipid Peroxidation Chain Growths

**Figure 1:**
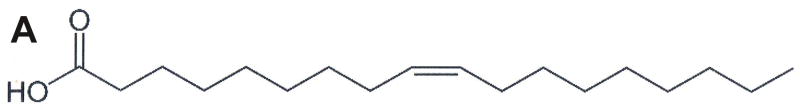

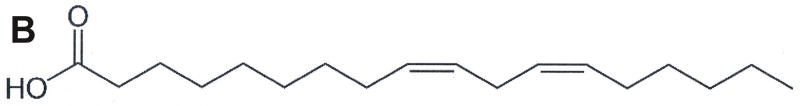
Fatty Acids. (A) Oleic Acid with one C=C bond (B) Linoleic Acid with two C=C bonds.

**Figure 2:**
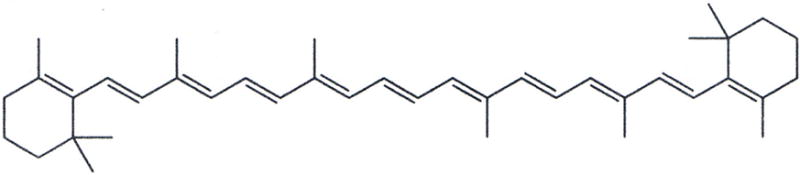
β,β-carotene conjugated C=C bond π system as two halves of the molecule for vitamin A.

Fundamental free-radical polymer chemistry with reactions through chain-growth polymerization by crosslinking across the unsaturated C=C bonds of low viscosity oils can subsequently form solid extremely large macromolecular rubbery gels [1,49]. Representative molecules for plasma cell membrane unsaturated lipid fatty acids are presented as oleic acid, Figure 1A, and linoleic acid, Figure 1B. Also, another highly unsaturated biomolecule with β,β-carotene that can be dissociated into two molecules of vitamin A is shown in Figure 2.

**Figure 3:**
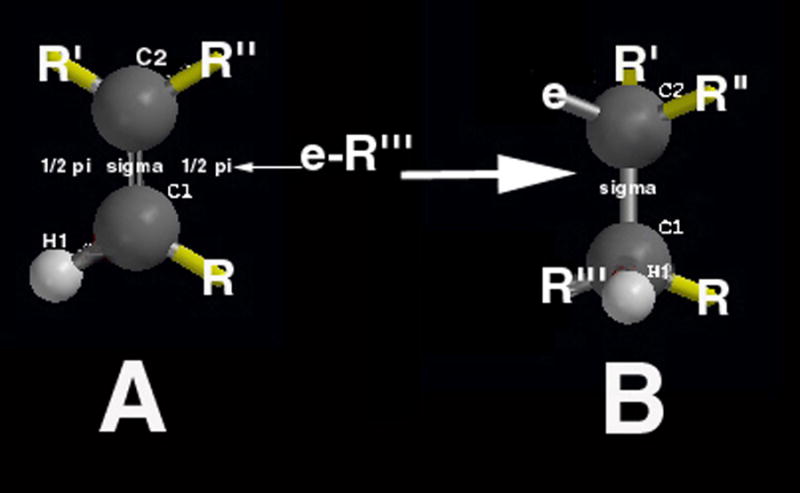
(A) An unsaturated molecular group with one C=C π-bond is attacked by a highly unstable free radical as e-R‴ to form a covalent sigma bond on one carbon atom and through a reactive secondary sequence create (B) all single bonds as a more saturated molecule with an unstable free radical on the opposite carbon atom. (Micromechanics/Electron Interactions for Advanced Biomedical Research (2011) Chapter 16 Free Radical Reactive Secondary Sequence Lipid Chain-Lengthening Pathologies. Figure 5. Richard Petersen and Uday Vaidya).

During free-radical unsaturated lipid chain-growth as unsaturated C=C bonds are replaced by saturated carbon-carbon single bonds the carbon atoms on each side of the double bond are oxidized to lower electron densities. Consequently, polyunsaturated phospholipids of the plasma cell membrane with one or more C=C bonds are particularly susceptible to a free-radical oxidation process [35] changing from a low-viscosity oil to thicker oil and even to a solid product [1]. A free radical is an unstable reactive species containing an unpaired electron in a molecular orbital that can initiate addition reactions on either side of the C=C pi (π) bond [1,50]. The C=C π bond is an exposed orbital that is readily attacked by an electrophile in free-radical form to produce a σ bond on one carbon atom thus forming a free radical on the opposing carbon atom that can then react with another C=C π bond [1,50], Figure 3. The free-radical C=C π-bond reaction is exothermic so that the new σ bonds of the product are more stable than the original C=C π-bond of the reactant. Unsaturated C=C π-bonds can then continue to react continuously from one unsaturated lipid to another throughout a solution at some minimal free-radical concentration to transform a low-viscosity oil into a higher viscosity oil and even become a fully polymerized solid [1,49]. Increasing the number of C=C bonds increases unsaturated reactivity toward chain-growth polymerization when comparing oleic and linoleic acid with one and two C=C double bonds respectively to vitamin A and beta carotene with five and eleven C=C double bonds respectively [1,49].

**Figure 4:**
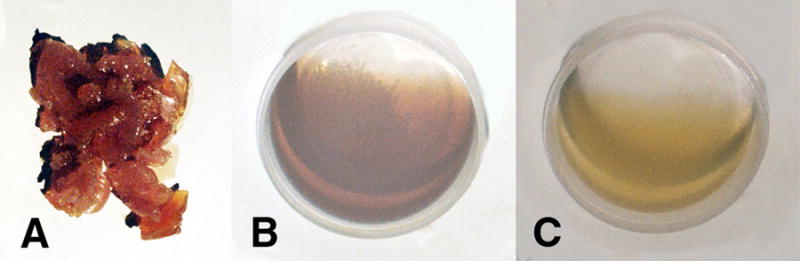
(A) Unsaturated fatty acid lipid oils, benzoyl peroxide free-radical initiator, cobalt naphthenate transition metal accelerator and acrolein α,β-unsaturated lipid aldehyde breakdown product crosslinker polymerized into solid rubbery gel. (B) Unsaturated fatty acid oils, benzoyl peroxide and cobalt naphthenate accelerator remain unreacted low-viscosity oil without acrolein crosslinker. (C) Unsaturated fatty acid lipid oils, benzoyl peroxide, and acrolein α-β unsaturated lipid aldehyde remain unreacted low-viscosity oil without cobalt metal free-radical accelerator. (Micromechanics/Electron Interactions for Advanced Biomedical Research (2011) Chapter 16 Free Radical Reactive Secondary Sequence Lipid Chain-Lengthening Pathologies. Figure 10. Richard Petersen and Uday Vaidya).

**Figure 5:**
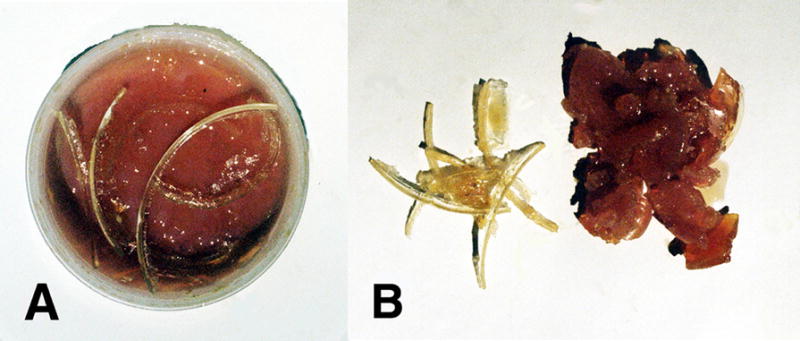
Comparison between free-radical polymerized reaction products for lipid peroxidation across O=O bonds and unsaturated lipid reactive secondary sequence polymerization along C=C bonds. (A) Reactive secondary sequence polymerization with crosslinker and unsaturated lipids form solid rubbery gel on the bottom. Crystalline polymerization products were pulled off the sides of the reaction container from acrolein crosslinked lipid and atmospheric oxygen that appeared to concentrate at the nonpolar polyethylene container surface. (B) Mass volumes compared between Left Side-crystalline lipid peroxidation polymerization products of acrolein crosslinked lipids and oxygen and Right Side-reactive secondary sequence polymerized unsaturated lipids solid rubbery gel. (Micromechanics/Electron Interactions for Advanced Biomedical Research (2011) Chapter 16 Free Radical Reactive Secondary Sequence Lipid Chain-Lengthening Pathologies. Figure 12. Richard Petersen and Uday Vaidya).

**Figure 6:**
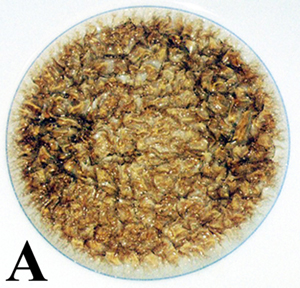

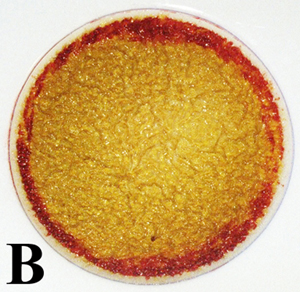

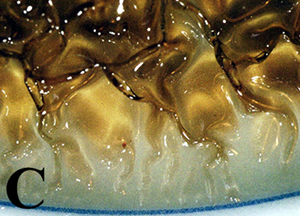

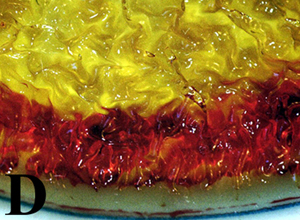

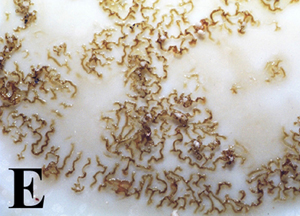

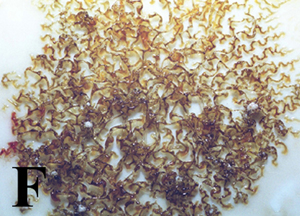
Free-radical polymerization from oil liquid to rubbery solid. Left Side: Vitamin A. Right Side: β,β-carotene. (A) Vitamin A solid rubbery gel low magnification. (B) β,β-carotene solid rubbery gel low magnification. (C) Vitamin A solid rubbery gel higher magnification. (D) β,β-carotene solid rubbery gel higher magnification. (E) Vitamin A solid rubbery gel high magnification for thin film. (F) β,β-carotene solid rubbery gel high magnification for thin film. Thin films in Figures 6E and 6F were exposed to higher concentrations of air O_2_ that amplified the O=O bonds for the free-radical lipid peroxidation crosslinking and created extensive cure-shrinkage spaces between the solids formed. (Micromechanics/Electron Interactions for Advanced Biomedical Research (2011) Chapter 16 Free Radical Reactive Secondary Sequence Lipid Chain-Lengthening Pathologies. Figure 16. Richard Petersen and Uday Vaidya).

With benzoyl peroxide free-radical initiator and a cobalt cation transition-metal free-radical accelerator, mixtures of unsaturated oils containing 90:10 wt% oleic/linoleic acid turned to solid rubbery gels by free-radical chain-growth polymerization, Figures 4 and 5, only when α,β-unsaturated lipid aldehyde breakdown products that acted as crosslinking agents such as acrolein (CH_2_=CH-CHO) or crotenaldehyde (CH_3_-CH=CH-CHO) were added [1,49]. On the other hand, nutrient capsules with oils having vitamin A or beta carotene polymerized into solid rubbery gels by free-radical polymerization, Figures 6A–D, without the need of acrolein or crotenaldehyde free-radical crosslinkers [1,49]. Alternatively, isoprene, C_5_H_8_ (CH_2_=CH-CH_2_-CH=CH_2_) with two end-group vinyl C=C double bonds would polymerize by free-radical chain growth reactions without the need of an unsaturated aldehyde crosslinker [49]. As a fundamental property, isoprene has two C=C vinyl end groups to account for increased reactive secondary sequence crosslinking into a macromolecular solid. Regarding crosslink capability, acrolein free-radical reactivity is much greater than crotenaldehyde [49,51] because the unsaturated acrolein has an easily accessible vinyl C=C substituent end group, but the unsaturated crotenaldehyde C=C group is internal [49]. Acrolein reactivity is 100–150 times greater than crotenaldehyde and 4-hydroxynonenal, another α,β-unsaturated aldehyde lipid breakdown product [51,52,53]. In related opposite oxidation lipid chemistry, unsaturated lipid fatty acids from cellular membranes can break down to lower molecular weight molecules through oxidation to form aldehydes [1] to include acrolein, crotenaldehyde, 4-hydroxynonenal and numerous other α,β-unsaturated aldehydes such as 4-hydoxyhexenal and malonaldehyde [51,52,54,55,56]. Also, during oxidative stress acrolein has been shown to strongly crosslink proteins [57].

**Figure 7:**
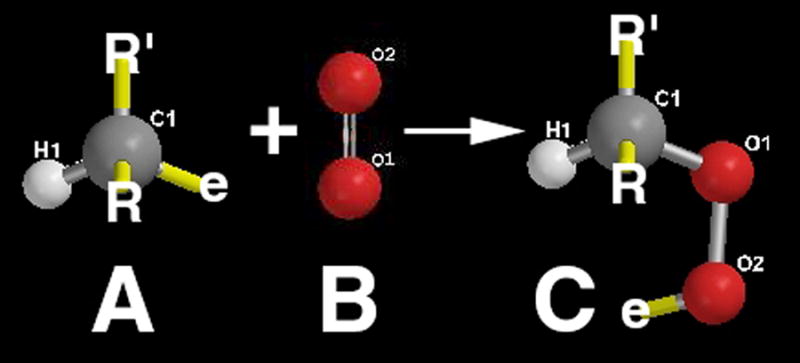
The lipid peroxidation from a saturated lipid -CH_2_- backbone group after hydrogen abstraction or attack on an unsaturated lipid C=C bond by a free radical to form (A) a carbon-centered lipid free radical and then subsequent combining with (B) O_2_ and the O=O π bond thus forming (C) a lipid peroxyl free radical with large reactive electrophile swing rotation by two O_2_ σ single bonds. (Micromechanics/Electron Interactions for Advanced Biomedical Research (2011) Chapter 16 Free Radical Reactive Secondary Sequence Lipid Chain-Lengthening Pathologies. Figure 2. Richard Petersen and Uday Vaidya).

In terms of additional free-radical solids produced, unsaturated oil as 90:10 wt% oleic acid/linoleic acid with the acrolein crosslinking α,β-unsaturated aldehyde lipid breakdown product could generate gross free-radical lipid peroxidation chain-growth polymerization hard thermoset-type crystalline solids by chain-growth reactions with molecular oxygen double (O=O) bonds [1,49], Figures 5 and 7. Molecular oxygen is nonpolar and expected to concentrate along the sides of the highly nonpolar plastic polyethylene circular reaction well by similar nonpolarity attraction to provide secondary sequence O=O bonds during the crystalline free-radical reaction [1,49].

Incorporation of 3% dibenzoyl peroxide initiator and 4% cobalt naphthenate accelerator slow over a three-day period in 4.0 mm deep wells to both low-viscosity vitamin capsules as unsaturated oils with either vitamin A or β,β-carotene sequentially produced a thicker viscosity until both oil mixtures started to gel. Subsequent extended polymerization to solids with ruffling was complete at 12 days, Figure 6A–D photos. Thin films approximately 0.5 mm in depth of both vitamin A and β,β-carotene with cobalt naphthenate and dibenzoyl peroxide free-radical redox agents exposed to relatively large concentrations of atmospheric oxygen polymerized through reactive secondary sequence C=C bonds with volumetric shrinkage to disconnect by wrinkling upward and creating elevated ribbons as warped ruffling patterns, Figure 6E–F. Further, the thin film rubbery solids exposed to more oxygen were much harder, stronger and stiffer than solid rubbery gels polymerized in the disks about 4.0 mm deep presented in Figure 6A–D.

## 3. Mechanomolecular Theory for Free Energy Movement

**Figure 8:**
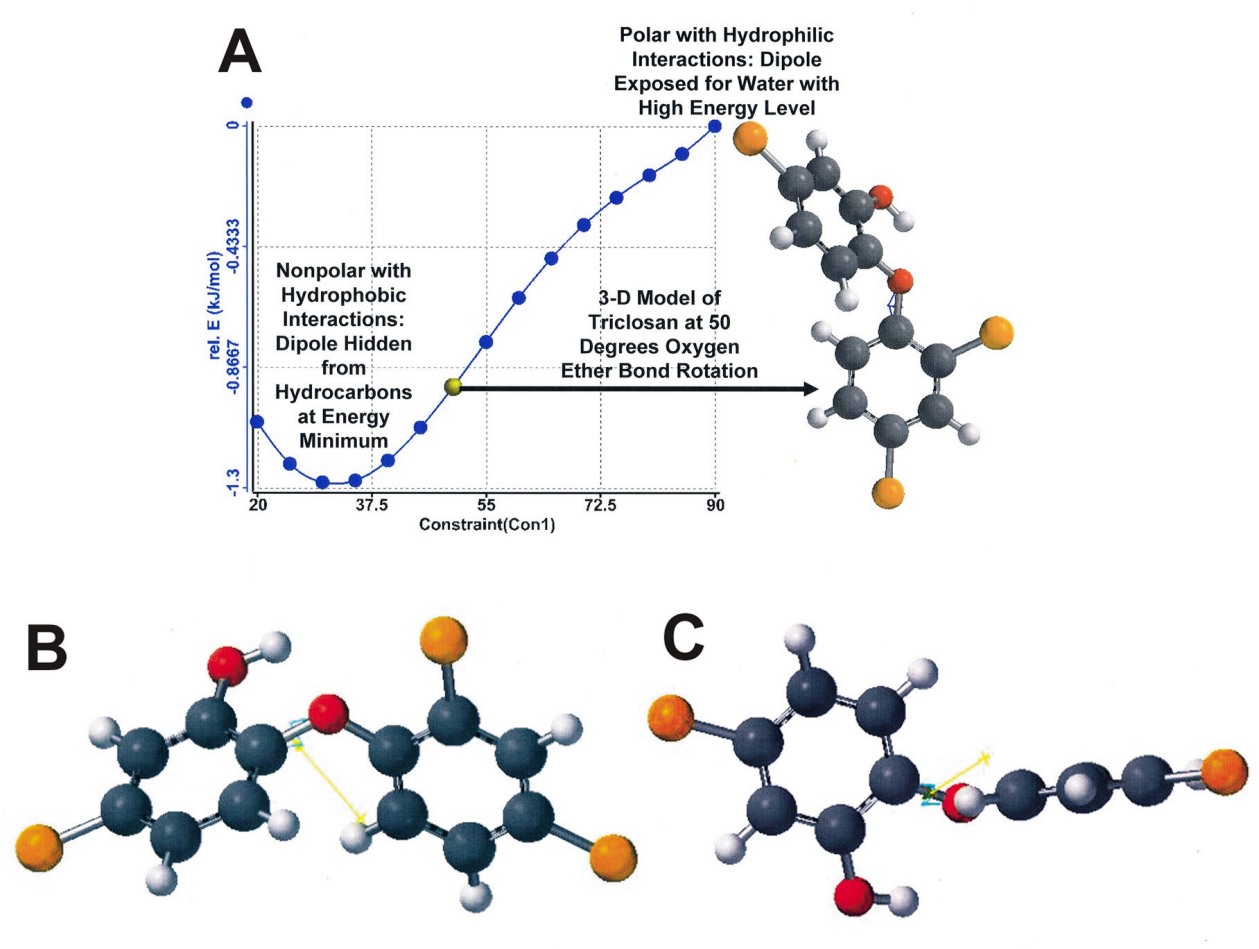
(A) The energy summary for the molecule triclosan is charted with bond rotation around the oxygen ether atom comparing the comparative bond energies between 20° to 90° bond rotation that gave an energy minimum at approximately 30*°* bond rotation. (B) Bond angle of about 30° at the energy minimum conceals the oxygen ether atom and lone-pair electrons with the dipole moment also hidden between aromatic rings. (C) 90*°* bond rotation of both aromatic rings around the oxygen ether bonds shows how oxygen lone-pair electrons are exposed with dipole moment equivalent to the high bond energy value. (Micromechanics/Electron Interactions for Advanced Biomedical Research (2011) Chapter 14 Mechanomolecular Computational Chemistry Theory with Triclosan Models. Figure 1A–C. Richard Petersen, Jack Lemons and Michael Reddy).

Mechanomolecular Theory is a newer field of chemistry that can explain free molecular movements due to bond conformational changes accentuated by atoms containing at least one set of nonbonding lone-pair electrons [43]. Lone-pair electrons have bond energies that can change dramatically according to the molecular bond angles and positions of the associated bond atoms with a shift in response to the polarity of the immediate nano and microenvironments [43]. In nonpolar or hydrophobic environments bond angles adapt to hide lone-pair electrons whereas in a polar or hydrophilic environment bond angles adjust to expose lone-pair electrons, Figure 8 [43]. Computational restricted Hartree-Fock self-consistent field (SCF) method calculation was performed using Pulay DIIS extrapolation by Wavefunction Inc., Irvine, CA on Spartan Quantum Mechanics Module software for the triclosan molecule [43]. Instantaneous normalized unbalanced bond energy states at a nonpolar/polar boundary interact at fluctuating rapid rates. As a consequence, at the respective biologic fluid/cell membrane interface the combined hydrophilic/hydrophobic microenvironments can result in unstable polar/nonpolar bond conformation interactions with nonbonding lone-pair electrons [43]. Because bond energies cannot attain perfect equilibrium at such an accentuated different medium interface through changing mechanomolecular movements, during molecular repositioning shifts bond angles must change rapidly to hide and expose the lone-pair electrons at close simultaneous levels as part of the continuous free molecular movement. The end result is constant vibration at various levels of amplitude and distances from the diverse molecular-membrane complexes.

Protein movements were described with the use of x-ray crystallography as early as 1989 due to conformational bond rotational changes because of alternating vibratory action [41,42]. Important biologic atoms with lone-pair electrons that can provide mechanomolecular energy include oxygen having single-bond rotations and nitrogen with significant pyramidal inversions [43]. Vibration energy during bond fluctuations can better explain membrane transport with increased molecular diffusion, cell recognition, cell signaling, cell defense and enzyme mixing to speed reaction rates [43]. As a possible field of exploration, molecular bond vibration energy may prove a worthwhile method toward investigating molecular antioxidant effects related to improved oxygen diffusion or transport through cell membranes.

## 4. Calcium

Cell membrane organization can include amorphous or different forms of crystallized calcium phosphate to bond as molecular outer layers primarily on the extracellular phosphate head groups. Thus, calcium and phosphate increase structure that forms an outer layer veil to help seal and reduce permeability of the associated low-viscosity lipid fatty acid oils. Other compounds of interest for structuring the outer cell membrane layer to reduce permeability include calcium cements, calcium and magnesium thickeners and calcium carbonate to accelerate thickening and increase viscosity. Calcium (Ca^2+^) ions combine with the negatively charged cell membrane phospholipid groups [58,59]. Due to a much higher concentration of Ca^2+^ ions outside the cell than inside the cell [58,59,60], Ca^2+^ ions attach mostly on the outer membrane surface [58,59]. Bound Ca^2+^ ions do not influence the general membrane potential that is controlled by the diffusible ions for the Nernst potentials [58]. However, Ca^2+^ ions create light increased positive area surfaces on the outer membrane surface to produce a membrane hyperpolarization and reduce the excitability of close Na^+^ channels in the surrounding area [58,59]. Conversely, low serum or extracellular Ca^2+^ levels increase Na^+^ permeability with an influx to increase excitability, seizure, tetany and convulsions through membrane depolarization with an upward position of the membrane resting potential toward the cell firing threshold [58,59,61]. Hypercalcemia produces cardiac arrhythmia and lowers neuromuscular excitability [61]. Magnesium (Mg^2+^) another divalent cation influences the cell firing threshold similar to Ca^2+^ [59].

In terms of a material, calcium can cement in aqueous form as a hydroxide (Ca(OH)_2_), crosslink with water forming cement as calcium oxide (CaO), blend with hydrocarbons to pack by van der Waals forces of attraction as a carbonate (Ca(CO_3_)) and also form inorganic amorphous calcium phosphate (approximately CaPO_4_) broadly in soft tissues and blood vessels. Also, calcium is recognized as intercellular cement that helps to hold tight junctions together [61]. Inorganic calcium phosphate exists in complex compositions depending on the Ca/P ratio, Table 1 [47]. In terms of the physiologic property for pH and acid, HCO_3_^−^ is part of the most common extracellular buffer with carbonic acid [62]. Ca(CO_3_) sold as calcium carbonate is the most widespread particulate filler used in fiber-reinforced composites and molding compounds that further increases the viscosity of the hydrocarbon resin [46]. During the manufacture of fiber-reinforced composites, group II oxides and hydroxides like CaO/CaOH with MgO/MgOH are the most frequent resin thickeners that combine with carboxy-terminated end groups to form large coordinated complexes [45] that should apply to proteins and peptides attached to membrane lipids. Calcium hydroxide (Ca(OH)_2_) is dental cement used to protect the tooth pulp in deep fillings [48].

Calcium as a metal element is an excellent conductor of electricity [63], Table 2. On the other hand, membrane phosphate head groups help form a semiconductor at the interface with aqueous biologic fluids [64]. As a result, the finest elemental calcium atom-sized circuits could structure as fast electrical conductors overlying membrane/fluid interface semiconductors that aid to dissipate a damaging electron build up from the mitochondria electron transport chain during transient periods of hypoxia over more broad outer membrane surfaces. In related Biomaterial development, bone implant advances comparing a carbon fiber-reinforced composite to titanium-6-4 alloy were considered partially due to overall semiconducting properties of the total polymer matrix composite material combined with carbon fiber microbiocircuit electrical conduction having polymer insulation [65]. The polymer matrix carbon fiber-reinforced implant was considered to possibly provide safe efficient electron-transfer speeds at the osteocyte cell membrane level while rapidly removing excess damaging electron radicals that develop at a surgical implant site [65].

## 5. The Cell Membrane and Fatty Acids

### 5.1. Cell membrane organization of lipid and globular intrinsic proteins

**Figure 9:**
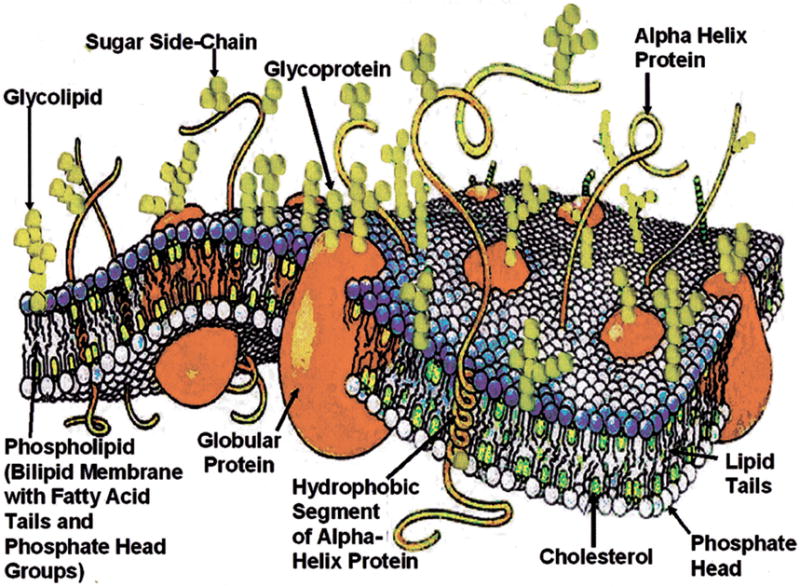
The fluid mosaic membrane model of a cell with intrinsic membrane proteins contained within and sugar groups modeled on the outer surface. (Micromechanics/Electron Interactions for Advanced Biomedical Research (2011) Chapter 14 Mechanomolecular Computational Chemistry Theory with Triclosan Models. Figure 12. Richard Petersen, Jack Lemons and Michael Reddy).

The current general F-MMM structure for a cell membrane describes a fluid lipid bilayer with globular proteins embedded [2,3], Figure 9. Cell membrane structural organization includes the lipid bilayer with proteins inserted within the bilayer or on the surfaces [2,3,70]. The model for random organization of the cell membrane has changed where membrane arrangement includes a range of domains with lipid-lipid and lipid-protein interactions that create explicit structural and functional properties [70]. Protein functions as enzymes are of particular cell physiologic significance [70]. Lipids and proteins are found in a large variety at an approximately 50:50 mass ratio [4]. But, since proteins on average are many times larger than an average lipid molecule, lipid molecules are about 50 times more numerous [4].

### 5.2. Lipids

**Figure 10:**
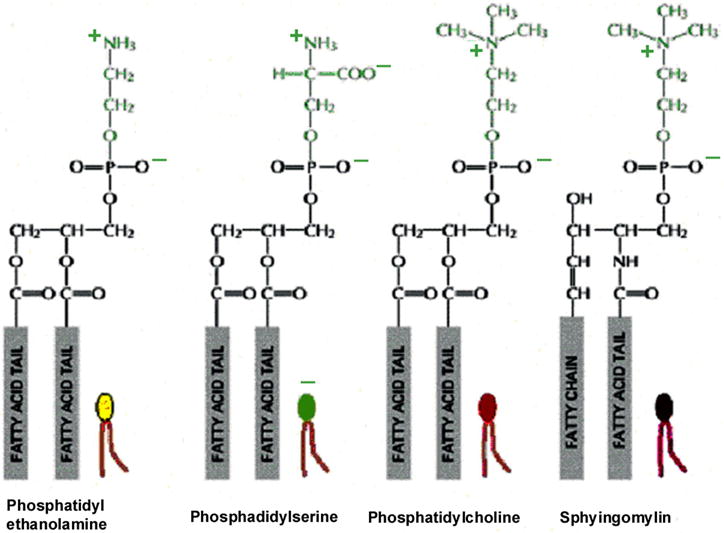
Most common mammalian membrane lipids. (Molecular Biology of the Cell. 4th edition. The Lipid Bilayer. Figure 10–9. Copyright © 2002, Bruce Alberts, Alexander Johnson, Julian Lewis, Martin Raff, Keith Roberts, and Peter Walter; Available from: http://www.ncbi.nlm.nih.gov/books/NBK26871/).

Lipids are nonpolar organic molecules with limited water solubility requiring organic solvents for extraction [71,72]. Lipids of most abundance in cell membranes are the phosphoglycerides that are amphipathic by containing both a double set of inner central hydrophobic (nonpolar) fatty acid tails linked to outer hydrophilic (polar) globular phosphate heads that can interact with water on both sides of the membranes [4,71]. The four most common lipids in mammalian membranes include phosphatidylethanolamine, phosphatidylserine, phosphatidylcholine and sphingomyelin [36], Figure 10. Consequently, as a result of the outer amine group attachments to the phosphate heads, oxygen dihedral single bond rotations and nitrogen inversions provide considerable capability for free mechanomolecular vibration on the outer membrane surfaces especially when combined with sugar attachments. Precise organization and control of membrane activities during lateral lipid movements are possible [70] particularly by the diversity of amine and sugar attachments with lipid polar phosphate headgroups for enzymes associated with the membrane. The membrane forms by stabilization of amphipathic properties between combined hydrophobic interactions of the fatty acid tails that exclude biologic fluids from the inner bilipid core connected with hydrophilic interactions of the outer phosphate headgroups that attract water [2,36]. Lipid fatty acids with cholesterol form the hydrophobic inner membrane core that rejects water so globular proteins of related overall molecular polarity can be similarly located within the membrane but with some normal repositioning movement [2,36]. Van der Waals forces of attraction between all molecules in the membrane provide added organization to support structural longevity [2,36]. Further, hydrophilic domains of the intrinsic proteins can extend as polar lengths from the membrane surface in the immediate nearby biologic fluids [2,36].

**Figure 11:**
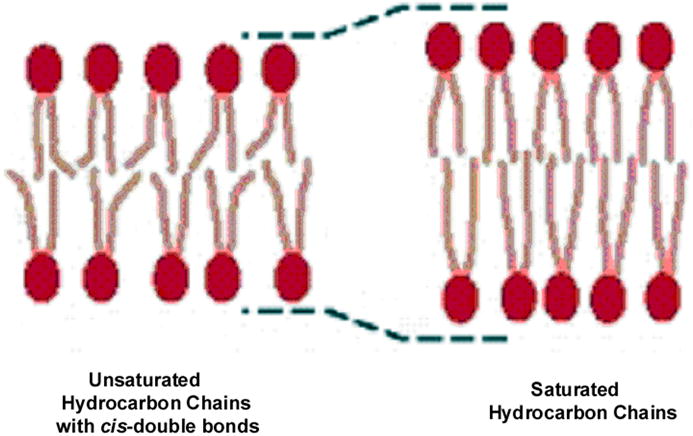
Cell membranes have asymmetry in part due to unsaturated fatty acid tails that kink and spread apart to shorten the membrane width and further increase fluidity (Left) while also containing saturated fatty acid tails that pack closer together and then extend chains longer in length and entangle to reduce fluidity (Right). (Molecular Biology of the Cell. 4th edition. The Lipid Bilayer. Figure 10–12. Copyright © 2002, Bruce Alberts, Alexander Johnson, Julian Lewis, Martin Raff, Keith Roberts, and Peter Walter; Available from: http://www.ncbi.nlm.nih.gov/books/NBK26871/).

Lipids are classified as fats or oils depending on the level of bond saturation [71,72]. As saturation is reduced by increasing the number of C=C bonds, solid saturated fats become low-viscosity unsaturated oils [71]. Unsaturated fatty acids in the cis configuration contain at least one planar non-rotating C=C bond that kinks the straight chain molecule outward and wider [36,71]. Consequently, unsaturated C=C bonds do not pack as closely together as more ordered saturated single bonds that further extend more rigid in longer length [36,71], Figure 11. Subsequent unsaturated fatty acids can move past one another more easily than saturated fatty acids with all rotating carbon-carbon single bonds that can entangle as molecules are packed more closely together [36]. Further, shorter fatty acid hydrocarbon tails are more fluid than longer hydrocarbon tails because shorter chains reduce bond rotation entanglements [36]. Related to C=C bond unsaturation, the melting point goes down with increased unsaturation that is a property related to increased fluidity of a cell membrane [36,71]. Further, increased lipid chain motion is manifested by decreased lipid viscosity, less membrane packing and lower lipid gel to liquid transition temperatures [73,74]. Also, the fluidity of a membrane is represented by measurements of globular protein rotations and movements that could even be extremely fast and lateral in the membrane [3].

## 6. Mitochondria and Production of ROS

### 6.1. Molecular oxygen for energy synthesis and ROS for pathology

During aerobic energy synthesis of the mitochondria, molecular oxygen (O_2_) with help from certain enzymes removes electrons and protons forming covalent bonds to make water [75]. O_2_ and H_2_O are considered conjugate redox pairs as one or more electrons or protons can be added to convert one molecule to the other [75] for example:

(1) $$½\;O_{2}+2e^{-}+2H^{+}\to H_{2}O$$

So, a 50:50 equimolar mixture ratio of O_2_ and H_2_O act as a conjugate acid-base pair for buffer capability to maintain H^+^ concentration or pH that expresses the logarithm to the base 10 of the reciprocal for H^+^ concentration [75]. Further, the O_2_ and H_2_O conjugate buffer pair maintain the electron concentration or redox (reduction-oxidation) potential [75]. The pH of pure water is neutral at 7.0 while the pH of arterial blood is 7.45 and pH of venous blood 7.35. If arterial blood pH drops below 6.8 or rises above 8.0 death will occur [76]. Further, the phosphate buffer system is a good intracellular buffer [76] and found with a large supply source on both sides of the bilipid membrane as phosphate head groups [4,36]. O_2_ is nonpolar and diffuses through similar nonpolar phospholipids in cell membranes to aid in the removal of the excess electrons and protons when energy is synthesized by mitochondria. However, during periods of intermittent hypoxia radicals can form by excess production of electrons from the electron transport chain and hydrogen cations can build from overload increase of the associated proton gradient. During the production of cell energy under standard physiologic conditions, mitochondria generate over 90% of the adenosine triphosphate (ATP) through oxidative phosphorylation for the cell [77,78]. Through such energy production mitochondria consume approximately 85% of the O_2_ used by the cell [26]. But, the general cause of concern during energy production is that mitochondria generate electrons and are the subsequent main cellular producers of free radicals as reactive oxygen species (ROS) like superoxide anion (O_2_^•−^) for the one electron reduction of O_2_ [26,27,39,79–83]. Related to cerebral ischemia mitochondria produce ROS that lead to cell death and overproduce ROS after return of O_2_ blood flow [84]. ROS include O_2_^•−^, hydrogen peroxide (H_2_O_2_) and the hydroxyl radical (HO^•^) [18,26]. Free radicals such as O_2_^•−^ and HO^•^ are unstable molecules with an unpaired electron. On the other hand, H_2_O_2_ is stable but can produce HO^•^ in the presence of a transition metal cation such as divalent iron (Fe^2+^) [1]. Subsequent ROS generated by mitochondria at high levels can then cause damage to lipids, proteins and DNA [12,13,18–24,84] and increase pathology [13,18,21,23,82] and even increase aging [20,25–28,80]. Conversely, ROS at low concentrations provide a level of biology for physiologic protection [12–18].

### 6.2. Protons and electrons of the mitochondrial inner membrane

**Figure 12:**
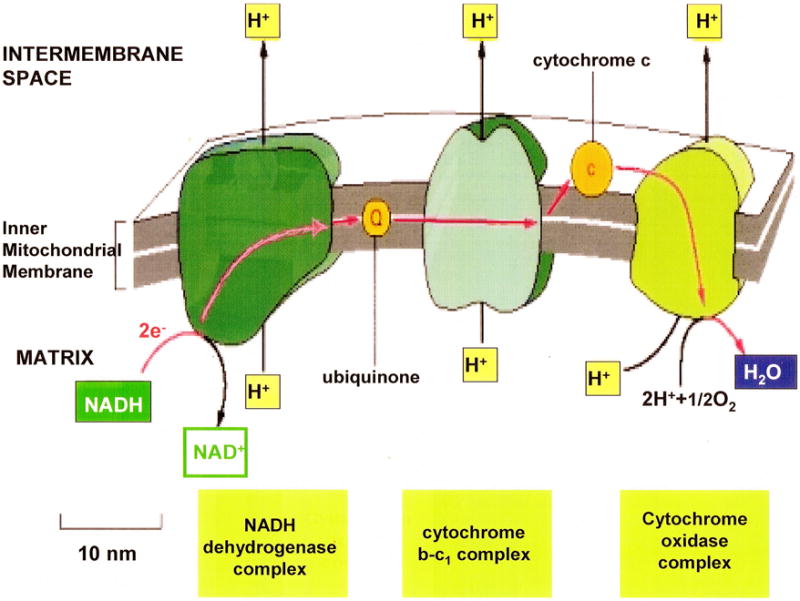
The mitochondrial proton gradient and electron transport chain require O_2_ at the last stage to remove electrons and protons and form water. (Molecular Biology of the Cell. 4th edition. Electron-Transport Chains and Their Proton Pumps. Figure 14–26. Copyright © 2002, Bruce Alberts, Alexander Johnson, Julian Lewis, Martin Raff, Keith Roberts, and Peter Walter; Available from: http://www.ncbi.nlm.nih.gov/books/NBK26904/).

The metabolic breakdown of complex carbohydrates, fats and protein molecules into small molecules liberates energy that is attached in adenosine triphosphate (ATP) and also produces coenzymes such as the reduced form of nicotinamide adenine dinucleotide (NADH) for additional oxidation energy release [85]. During aerobic metabolism for maximum energy production NADH is oxidized to NAD^+^ giving up 2 electrons and a proton, Figure 12. The proteins within the inner mitochondria membrane have some transport energy through mechanomolecular vibrations by fluctuating bond inversions and rotations particularly with electrons rapidly changing local membrane polarity [43]. Consequently protons can move through protein channels while simultaneously interacting with electrons that pick up and release protons while moving from one membrane protein to the next and pump H^+^ across a concentration gradient to the intermembrane space having less H^+^ protons [75]. On the other hand, electrons are passed along through the inner mitochondrial membrane by the electron transport chain as delocalized radicals with the globular membrane proteins in addition to cytochrome c as another globular protein and also moved on by the molecule ubiquinone that is reduced and oxidized [75]. At the end, the electrons are in the lowest energy state to then combine with protons and oxygen with help from other proteins specific as enzymes in the last water-forming reactions [75] that include formation of H_2_O_2_ as an intermediate. But, if oxygen is not available electrons and protons necessarily increase according to equation 1 that would contribute to ROS production and acid.

## 7. Globular Intrinsic Proteins

The globular intrinsic mobile proteins of the cell membranes should be characterized at some extent to appreciate the role for structural organization and unique biologic properties. As organizing elements, proteins may be structural constituents attached to the outer charged membrane surfaces or contained within the membrane [70]. Integral membrane proteins include a hydrophilic region on two ends and a larger hydrophobic region that extends through the lipid core [3]. So, smaller hydrophilic protein groups tend to be pushed toward water at the membrane surface and other polar groups while larger hydrophobic protein groups with stronger interactions are pushed away from biologic fluids at the membrane interface toward the nonpolar or hydrophobic lipid core [3,4]. Consequently, depending on the polarity of the immediate membrane microenvironment globular proteins can change conformation by folding to best adapt with the surroundings to either interact with or exclude from polar molecules as water or nonpolar molecules like fatty acids. As examples, nonpolar protein groups would interact with nonpolar fatty acid chains [3]. Conversely, intrinsic transmembrane globular proteins can have hydrophilic groups forced internally away from nonpolar bilipid fatty acids to form ion channels [3,4] that are selective in passing ions by connecting the cytosol with the extracellular biologic fluids. Subsequent ion channels then help maintain ionic concentrations and membrane electrical properties [3]. Further, proteins have mechanical energy to move and produce motion [41,42,43]. In addition, the largest groups of buffers in the biologic fluids are proteins as intracellular proteins and the plasma proteins [76]. Also, the most common antioxidants for ROS are identified as proteins by the enzymes superoxide dismutases, catalase and glutathione peroxidase [27,79,80,86] with radical delocalization by proteins into side chains and peptide bonds [87]. Buffering of acids and bases with ROS and the delocalization of electrons as radicals thus maximizes protein enzyme specificity to fold most effectively for substrate reactivity, provide mechanomolecular mixing motion for increased reaction rates and afford the possibility of overcoming large thermodynamic energy barriers for bond dissociation during chemical reactions. In terms of increasing bond dissociation rates, peptide bonds can be hydrolyzed with a strong acid or base that can be used at the same time by an enzyme since the rigid protein structure is able to prevent acids or bases from combining as in a biologic fluid [88]. However, due to enzyme specificity small changes in hydrogen ion concentration or pH influence the shape of enzymes that are proteins and thus enzyme activity to such an extent that reactions can be faster or slower [76]. Further, lateral mobility provided by cell membranes offers proteins the opportunity to best form enzymatic reactions depending on more exact locations of the substrate.

## 8. Reactive Secondary Sequence Crosslinking and Fluidity Relative to Unsaturation

The F-MMM provides for a detailed understanding regarding restrictions to lateral diffusion within a membrane of lipids and proteins [2,3]. The current model shows that the lateral mobility of proteins is dependent on membrane fluidity in addition to protein size or protein aggregation [70]. In a general chemistry description, fluidity within the restricted exclusivity of the membrane provides lateral mobility of lipids and protein molecules to seek other molecules with similarities in covalent polarities by attractive forces within the membrane proper. Also, lipids and proteins seek other molecules for comparable polarity with cytoskeletal filaments or the extracellular matrix. Further, polar protein pores can move toward those biologic fluids of most related polarity for better efficiency in transmembrane ion transport.

In terms of more cell membrane organization, cholesterol and cholesterol-protein can come together into lipid rafts [35]. Saturated rigid lipid regions that impose restrictions to fluidity associated with free-radical crosslink chemistry by ROS along with lipid rafts have been shown to increase with increasing H_2_O_2_ [35]. Membrane structural rigidity organization in the plasma membrane increases with the loss of fluidity during aging and also oxidative damage within the inner mitochondria membrane decreases fluidity [26]. Unsaturated fatty acids of the plasma cell membrane and in particular polyunsaturated fatty acids (PUFAs) are susceptible targets for electrophile free-radical ROS during lipid peroxidation [34,35] due to the exposed π orbital of the C=C bond that results in saturated lipid products [1]. Further, cell membrane cholesterol-rich lipid rafts play a major role in platelet aggregation [89]. Estrogen is related to cholesterol by nonpolar molecular structure similarities and during low concentrations of thrombin that aggregates platelets estrogen coordinates platelet aggregation through membrane estrogen receptor translocation to lipid raft domains only when sufficient cholesterol is present and not depleted [89]. In similar cellular recruitment nonpolar estrogen properties of the bisphenol polymer in a fiber-reinforced composite bone implant were considered a factor in significant statistical accentuated increases for bone formation related to organization by cell membrane attraction [65].

During lipid peroxidation of erythrocyte membranes that contain high concentrations of PUFAs fluidity is reduced where evidence of free radical injury is suggested [33,34]. Concurrently during erythrocyte membrane peroxidation, a reduction in PUFAs measured by six C=C bonds [33] could also occur during C=C free-radical crosslinking that also reduces fluidity with viscosity increases observed during unsaturated lipid oil fatty acid reactive secondary sequence chain growth to solids [1], Figures 4 and 5. Further, free radicals distort erythrocyte membranes by creating pointed extensions [34] that is characteristic of free-radical crosslinking in cancer cell membranes [87]. Therefore, free-radical crosslinking of membrane PUFAs during intermittent periods of hypoxia with low O_2_ mitochondria levels could account for loss of membrane fluidity or increased membrane rigidity and subsequent pathology. Even longevity is suggested to increase with lower levels of fatty acid unsaturation as a general characteristic because of less sensitivity to the risk for peroxidation of C=C bonds [90]. Also, oxidative damage that accumulates with age is considered significant in producing mitochondrial decay in aging [26]. During radiation exposure erythrocyte membranes increased saturated fatty acids over PUFAs with a loss of antioxidant enzymes catalase and glutathione peroxidase as indications of free-radical C=C bond crosslinking toward reduced fluidity [38]. But, as a quick measure of PUFA damage the determination of enzymatically catalyzed lipid aldehyde lower molecular weight breakdown products like malonaldhyde appeared to increase fluidity of the lipids over a 72 hour exposure [38]. Conversely, free-radical crosslinking by lipids with proteins decreased fluidity [38]. The first step in lipid peroxidation with a decrease in PUFAs might be a molecular breakdown into lower molecular weight products by a loss of C=C bonds to form aldehydes such as malonaldehyde that does not crosslink lipids [31]. But, other lipid aldehyde lower molecular weight breakdown products can form such as acrolein and crotenaldehyde [51,52,54,55,56,91] that help to form free-radical crosslinks with unsaturated C=C lipid bonds [1]. The loss of PUFA C=C bonds could account for a decrease in molecular motion with increased lipid order by covalent crosslinks despite a measured increase in the lower molecular weight lipid breakdown product malonaldehyde [31]. As an alternate example, the endoplasmic reticulum is an intracellular membrane located near the cell nucleus that has a brief loss of fluidity after initiation of free radicals [32]. Also, in diabetes the cell membrane ratio for PUFAs to saturated fatty acids decreases with an increase in membrane stiffness [37] suggesting a loss of unsaturation by C=C crosslinks. Because the pathologic nature of diabetes is associated with ROS [13], the resultant ratio change of lower unsaturated to saturated lipids with increased membrane stiffness [37] is a sign of lipid free-radical C=C bond breakdown into aldehydes with loss of unsaturated lipids. Subsequent formation of lipid breakdown aldehydes can then also result in development of unsaturated aldehyde crosslinker products generated toward free-radical lipid C=C bond chain-growth covalent structural rigidity [1] that could increase membrane stiffness in diabetes [37]. As a major concern, lipid free-radical crosslinked membranes with reduced fluidity would increase the obstacle to oxygen diffusion and associated disease.

## 9. Free-radical Crosslink Pathology in Cancer Cell Membranes

**Figure 13:**
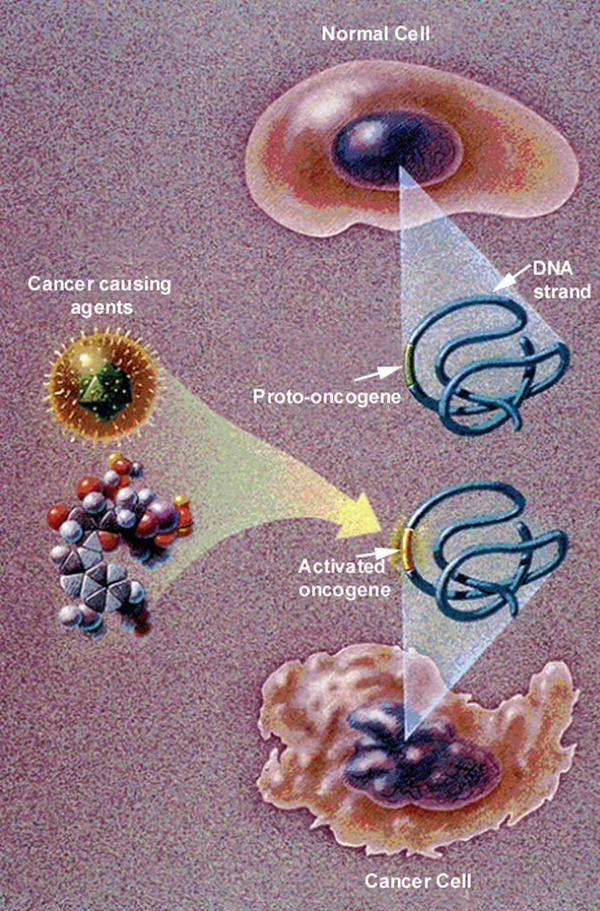
National Cancer Institute example for oncogene activation of a normal rounded cell with smooth nuclear and plasma cell membranes converting to a cancer cell with irregular nuclear and plasma cell membranes. (With permission from the National Institutes of Health/Department of Health and Human Services).

Mitochondrial production of ROS at low homeostatic concentrations has been considered physiologic on the cellular level [12–18]. For another explanation in ROS homeostasis with cell membranes, regular concentration levels of lipid oxidation breakdown products and ROS that crosslink PUFA C=C bonds might improve cell membrane organization with structural adjustments during normal ROS production by mitochondria. However, at higher pathologic ROS concentrations cell membrane fluidity is decreased toward more rigid structure [26,31,33,34,35,38]. High concentrations of free radicals are generated during hypoxia in cancer cells [87,92–100], predicted as following intermittent O_2_ supply during energy synthesis in the mitochondrial electron transport chain to initially produce O_2_^•−^ as the one electron reduction of O_2_ [26,27,39,79–83]. Cancer cell morphology represents an emphasis on oxidative stress that is particularly evident with the membranes [87]. Typical National Cancer Institute representations for cancer cells emphasize uneven borders with membrane ruffling and irregular-shaped nuclei compared to the membranes of normal smooth rounded cell with smooth oval nuclei [87], Figure 13.

A major characteristic of free-radical covalent bonding during chain-growth polymerization of a low-viscosity liquid-like material to solid structure for increased modulus and density is linear/volumetric cure shrinkage [1,46,101–104]. Free-radical cure shrinkage also produces possible warpage as one other distinguishing problem of extensive polymer electron-pair covalent bonding [1,46,104]. As shrinkage is not generally uniform with inhomogeneous material during covalent electron pairing by polymerization molecular chain growth in addition to increased modulus residual internal stresses are produced with warpage that weakens the set polymer [103,104]. Warpage in a cured polymer is heightened especially during free-radical polymerization with thin film coatings of uneven thickness without smooth underlying support [104]. As covalent bonds increase with σ bonds replacing C=C π bonds during free-radical reactive secondary sequence chain-growth polymerization, polymer chains come closer from more remote van der Waals intermolecular attraction forces so that bulk material is reduced by linear/volumetric cure shrinkage [1,101,102,103]. Free-radical C=C bond conversion to σ single bonds is a thermodynamically favorable reaction and forms an exothermic polymerization so that the new bonds of the product are more stable than the original bonds of the reactants [1]. As a result, unsaturated lipid oils that crosslink by free-radical chain-growth polymerization through reactive secondary sequence can produce linear/volumetric cure shrinkage without the need for extra energy introduced [1,46,101–104].

Actin fibers of the cytoskeleton confer strength and underlying support to the membrane of the cell [105]. The plasma cell membrane with unsaturated lipid oils and phosphate groups would then provide an environment that maintains a separate medium from the stronger actin fibers irregularly set to unevenly reduce free-radical lipid polymerization warping particularly as an inner thin support. Hydrocarbon lipid molecules drawn together at a rounder border would require some invagination to wrinkle inward especially when combined with coupling to intermittent underlying rigid actin fibers resulting in the explanation for common irregular membrane appearances of cancer cells depicted in the consensus National Cancer Institute illustration, Figure 13.

**Figure 14:**
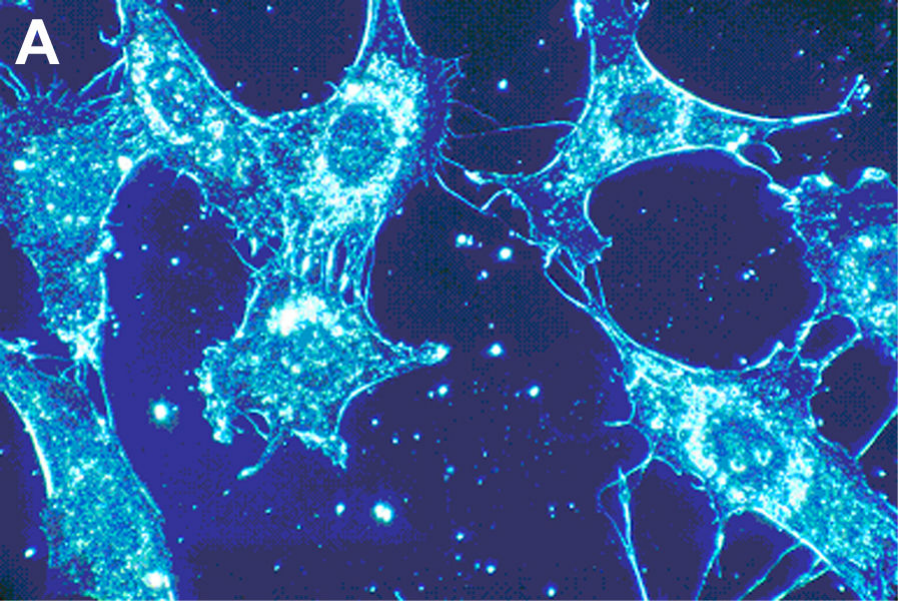

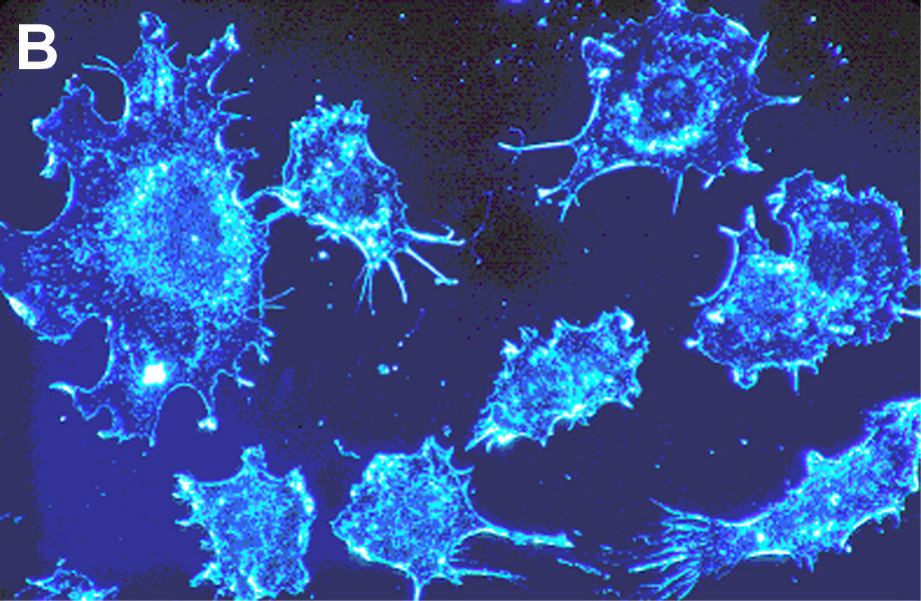
Cell cultures from human connective tissue 500× (A) Normal cells with smoother membrane borders. (B) Cancer cells with more spike-like protrusions revealing more irregular deeper plasma cell membrane invaginations. (With permission from the National Institutes of Health/Department of Health and Human Services).

Similar to cancer cell irregular membrane borders, highly unsaturated oils in nutrient capsules with numerous C=C bonds as vitamin A and β,β-carotene for free-radical crosslinking and polymerization cure shrinkage has previously demonstrated extensive wrinkling and warpage during a solidification polymerization process [1], Figure 6A–F. As well as irregular plasma cell membrane borders on the outer periphery of a cancer cell, the nuclear membranes for cancer cells are misshapen and nuclear to cytoplasm ratios increase [106]. Regarding C=C bond polymerization shrinkage with warpage, cell cultures that show normal cells with smoother membrane outlines compared to cancer cells with more irregular membranes include additional plasma cell membrane spike-type extensions that form deeper invaginated irregular borders as part of the transformation to cancer, Figure 14.

## 10. Invasive Cancer Cell Movement

**Figure 15:**
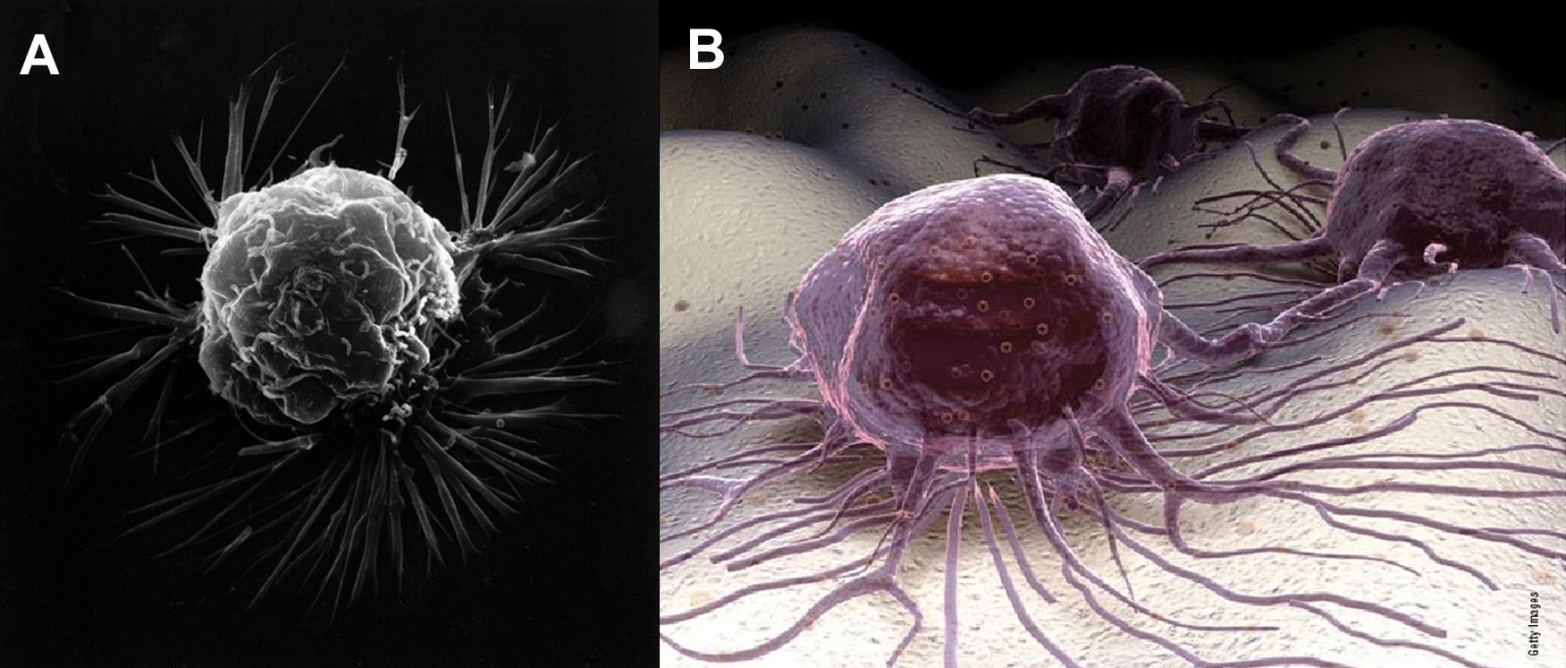
(A) SEM with membrane ruffling and long lamellipodia spike-like extensions. (B) 3D enhanced SEM image of cancer cell with membrane lamellipodia spike-like extensions on a tissue surface. (With permission from the National Institutes of Health/Department of Health and Human Services).

Cancer development involves cell movement through epithelial-mesenchymal transition (EMT) with changes in the cell shape and invasion of adjacent tissue [105,107]. Cancer cells have been shown to move in response to ROS such as H_2_O_2_ that can breakdown into hydroxyl free radical (HO^•^) and create projections at the cell membrane edges [108,109]. Cell motility can have controlled direction with attraction toward extracellular gradients of molecular intermediaries by a process termed chemotaxis [110]. ROS with H_2_O_2_ have promoted chemotaxis to control chemoattractants that attach to the cell membrane with actin polymerization for cell movement toward H_2_O_2_ and other ROS [111,112,113]. A cell develops membrane projections with adhesive attachments to the extracellular matrix capable of contracting as molecular bonds form to pull the cell forward [108,114,115]. The long projections extending outward from the plasma cell membrane are lamellipodia and the short focal adhesions are filopodia that are generated from polymerizing actin fibers at the advancing forward cell membrane edge [108,115]. On the rear cell membrane edge depolymerization of actin occurs to release the membrane adhesive attachments [108,115]. Delocalization of electrons from the mitochondria during oxidative stress appears possible through microtubules and actin fibers [87]. Actin fiber polymerization extending outward from the plasma cell membrane creates projections that hold focal adhesions in filopodia to the extracellular matrix and pull the cell forward as the adhesive bonds contract [108,114,115]. After actin fibers release at the rear cell membrane edge of the cell movement direction, actin molecules can be reused at the forward cell membrane edge for actin fiber polymerization assembly for advancing movement [108,115]. Cancer plasma cell membranes are shown by scanning electron microscopy (SEM) of different quality for a three-dimensional perspective on the irregular membrane borders with ruffling and wide spike-like projections lengthening away from the cell, Figure 15.

Covalent bond free-radical crosslinking and weaker secondary bonding provide a contraction process to bring large macromolecular structures closer together [1,101–104] that enable a cell to move forward. In addition, a PUFA with 6 C=C bonds has demonstrated a reactivity as a chemoattractant to initiate cell migration with lamellipodia actin fiber polymerization by using a strong free-radical initiator [116] with dominant free-radical polymerization capability through C=C bonds for covalent polymerization shrinkage [1] and possible forward cell contraction movement. Further, proteins can agglomerate or crosslink especially by the amino acid tyrosine [6,117] with cation transition metal-catalyzed reactions [6] as a second mechanism for forward bond contraction in cell movement.

## 11. Metastasis

**Figure 16:**
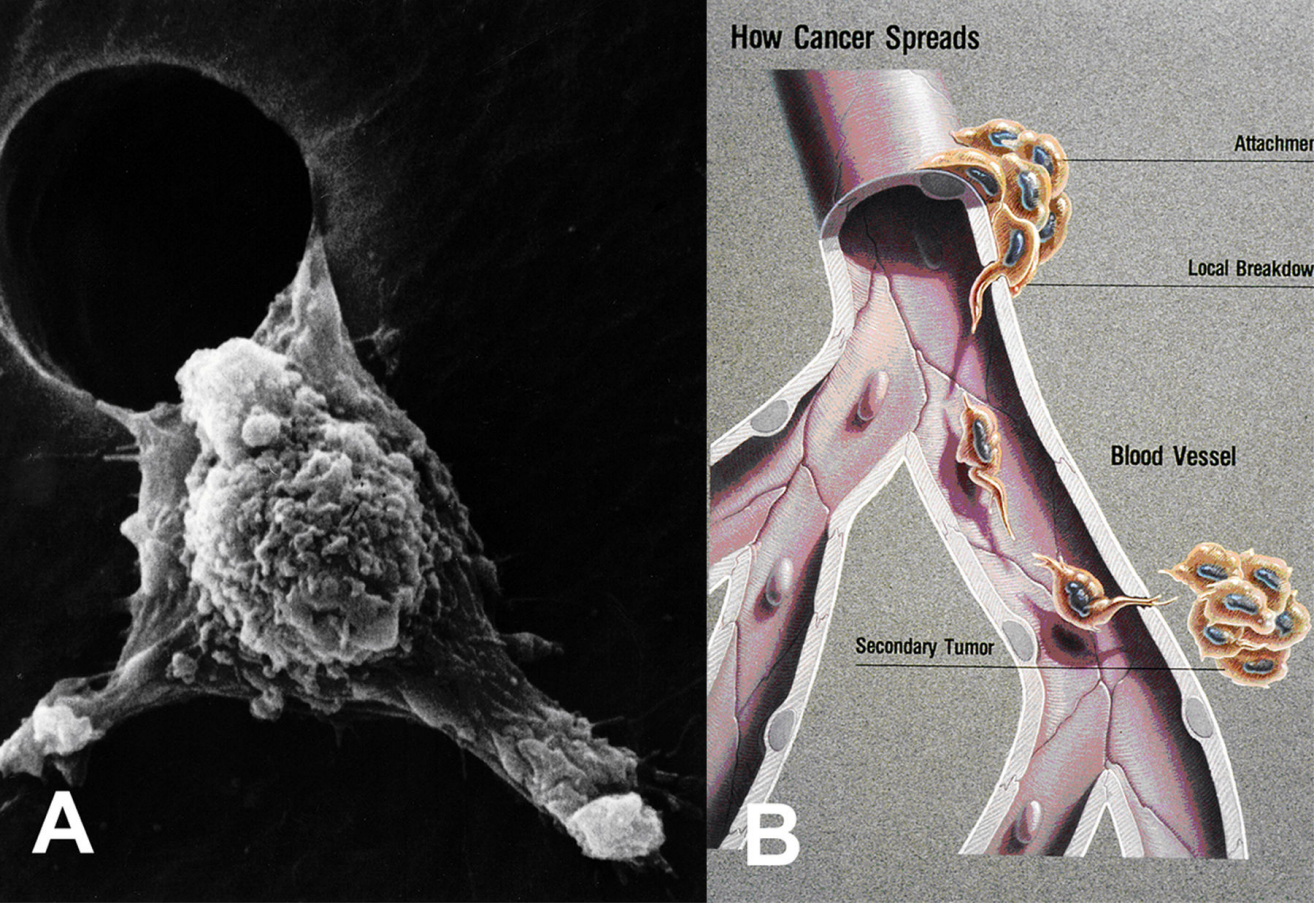
Metastasis (A) SEM of cancer cell moving through an artificial hole showing pseudopodia call lamellipodia. (B) Illustration shows how cancer cells attach to the blood vessel wall basement membrane, break down the blood vessel wall with lamellipodia extensions to enter the blood stream and move through the body with subsequent metastatic tumor formed at a distant site. (With permission from the National Institutes of Health/Department of Health and Human Services).

Although spike-like extensions of the cancer plasma cell membrane could greatly obstruct movement or leakage through small tissue spaces, invasive cancer cells have smaller membrane surface areas that accommodate such lower modulus cells to easily squeeze through small openings in the endothelium of blood vessels [118]. Still, spike-like extensions are prominent on cancer cells at the leading edge during metastatic movement [119]. Actin fibers orient along the axis of the lamellipodia extensions for the highest modulus to resist sideways deflection in the forward direction [119]. Consequently, once the lamellipodia squeeze through a small space leverage can be applied to the opening to deform the sides apart and invade into new tissue [119]. Figure 16 shows how low modulus cancer cells invade with long higher modulus lamellipodia extensions. Cytoskeleton fibers conduct electrons polarized from the negative centrosome near the nucleus to the positively charged outer plasma cell membrane side as radical negatively charged electrons to provide the chemistry for advancing actin fiber polymerization [87]. Electrons conducted through microtubules to actin fibers [120] are overproduced by mitochondria under oxidative conditions combined with hypoxia [87]. Actin has demonstrated restructuring when exposed to free radicals from H_2_O_2_ to enhance cell movement [121]. Regarding ability to start free-radical polymerization, H_2_O_2_ has been shown to be an excellent initiator in resin polyesters [122]. Through similar free-radical ROS chemistry oxidized low density lipids have been shown to cause actin polymerization in macrophages [123]. Also, H_2_O_2_ with other ROS are found in many cancer cells [92–100,124].

With metastasis cancer cells exhibit a lower modulus and lower viscosity than normal cells to deform more while also showing pleomorphic smaller sizes with less membrane area [125,126,127]. Cell stiffness or approximately modulus increases with organization of actin fibers of the cytoskeleton, but during cancer transformation actin fibers become more disorganized into irregular complexes so the cells become less stiff and distort more [127]. On the contrary, tissue tumor density increases that is a risk factor for cancer [128,129,130]. Increased stroma density is associated with more collagen deposition [128] that gives better traction forces for cancer cell membrane focal adhesions to encourage cell movement during metastasis [130,131]. Further, cells are inclined to move toward stiffer substrates [131]. In terms of membrane structure and organization, cancer cell pseudopods with high modulus actin fibers supply stiff leverage to travel through narrow spaces and invade adjacent tissue, Figure 16. However, by reverse structural organization lower extracellular pH seen with cancer cells activates protease enzymes to break intercellular adhesions of the membranes that allow a cancer cell to release from the main tumor [105] Also, protease enzymes create space for cancer cell invasion [105]. As a result, smaller cancer cells with lower moduli are allowed to leave the primary tumor and move through small gaps [118] such as openings in the endothelium caused by protease enzymes and enter the blood stream. However, larger cancer cells that eventually go into the blood vessels most often become trapped in the capillaries of the lung to metastasize into tissue while smaller cancer cells can metastasize to distant tissues [105].

## 12. Vitamin Supplement Clinical Trials

The Free-Radical Theory of Aging (FRTA) states that aging is the result of accumulative damaging changes that increase disease and death [25,28]. The chemical basis was considered a result of free radicals normally generated by mitochondrial oxidative enzymes during energy synthesis and the subsequent cation transition metal catalysts in the connective tissue [25,28]. Once produced free radicals reacted within cells and tissue to start the aging process [25,28]. Further, the FRTA suggested that chemical means could be initiated by antioxidants to provide protection from free radicals to decrease aging, increase lifetime and be successful in preventing disease [25,28]. An antioxidant is a molecule that scavenges free radicals to prevent damage to other molecules. Epidemiological studies indicated that nutrition particularly with fruits and vegetables with a relationship to antioxidants played a role in preventing diseases and prolonging life with vitamin A, beta carotene, vitamin E and vitamin C identified [132–145]. Age-associated diseases particularly susceptible to ROS included cancers, cardiovascular disease, neurological disorders and diabetes [134,139,142,144,145].

As a result of the many nutrition studies with diets rich in vegetables and fruit showing preventive results from disease, treatment was considered on the basis of possible vitamin antioxidant performance to counteract the detrimental effects of ROS. However, large vitamin supplementation studies using vitamin A and β-carotene, vitamin E or vitamin C or several combinations have not proven effective to prevent cancer [145–153] or cardiovascular events [147,148,154,155,156]. An alpha-tocopherol and beta-carotene cancer prevention (ATBC) clinical trial that included 29,133 male Finnish smokers with daily β-carotene (20 mg) for an average of 6.1 years statistically significantly increased risk of lung cancer 18% and overall mortality [146]. Daily vitamin E as α-tocopherol (50 mg) did not change the risk for cancer and had no effect on total mortality, but increased death from hemorrhagic stroke [146]. Total mortality for the ATBC study was 8% higher for men receiving β-carotene compared to placebo mainly because of lung cancer and ischemic heart disease [146]. Further, increased mortality in the ATBC study continued 4–6 years post-intervention [150]. A randomized beta-carotene and retinol efficacy trial (CARET) that examined a combination of β-carotene and vitamin A (30 mg) with smokers and asbestos-exposed worker found a statistically significant 28% increase in lung cancer compared to the placebo and 17% increase in total mortality rate that forced the trial to end 21 months earlier than designed [148]. In the selenium and vitamin E cancer prevention trial (SELECT), vitamin E as α-tocopherol significantly increased prostate cancer 17% [152].

Regardless of the disappointing clinical trial results with vitamin supplements, because diets high in fruits and vegetables containing vitamin A and vitamin E reduced risks for cancer and cardiovascular disease, beneficial properties may come from sources other than vitamins not yet identified or available in nutrient supplements [145]. As a major problem, antioxidant test results for covalent bond polymerization shrinkage with vitamin A and β-carotene both showed highly oxidative crosslink potential to turn low viscosity oils into solids when reacted with peroxide derived free radicals [1], Figure 6. Reducing related membrane fatty acid oil fluidity would interfere with oxygen diffusion and ultimately lead to increased generation of cellular free radicals during mitochondrial energy synthesis and associated diseases studied [13,21,23,24] whereby mitochondrial ROS increase pathology [18,82]. The lipid core of the vasculature with atherosclerotic plaque would also be susceptible to vitamin A and β-carotene free-radical crosslinking [49].

A few investigators believe vitamin E may not be stable enough to be an antioxidant but instead supplies alternate helpful properties for cellular function [157,158,159]. Further, during free-radical crosslink covalent bond measurements of fatty acids for inhibition by vitamin E to test the antioxidant potential, vitamin E provided no decrease at all in free-radical crosslink covalent shrinkage with only the noted observational benefit by reducing the viscosity of the lipids [1]. However, because vitamin E concentrates in cell membranes [157,159] increasing fluidity of the lipid fatty acids could increase oxygen diffusion as a related dietary antioxidant benefit not directly associated with pathological clinical manifestations of free radicals. Reduced lipid viscosity from vitamin E may help prevent various diseases by maintaining proper oxygen diffusion channels through cell membranes to account for mild preventive properties considered with nutritional dietary sources. But, once sufficient pathological crosslinked covalent free-radical structural barriers form, vitamin E appears unable to reopen oxygen channels to possibly explain vitamin E failure as a supplement during clinical treatments. In addition, an alternate adjunctive antioxidant role proposed for vitamin E requires protection complexed from other molecules that might be found in the diet [159,160].

## 13. Antioxidant Testing Problems and Solution

To better understand discrepancies between measured vitamin antioxidant potentials and clinical failure the primary vitamin antioxidant tests need consideration. Indices comparing different vitamins with activities of well-known antioxidants are based on electron transfer reactions between an antioxidant and the electrophiles that are free radicals requiring an extra electron for the unstable unpaired electron orbital [161]. The spectrophotometer method in the ultraviolet-visible light range is generally used to measure optical absorbance for free radicals and a known substrate with subsequent changes after an antioxidant such as a vitamin is added [161,162,163]. Fluorescence intensity is also measured [161]. Free-radical scavenging by the vitamin can then be compared for relative activity with other recognized antioxidants. Although a peroxide and a cation transition metal are the most common means to generate a free radical in a physiologic cell system, during most antioxidant spectrophotometer tests for radical scavenging chromogenic redox reagents with more stable free radicals are used that are colored [161,162,163]. However, optical changes based on color for maximum absorption with wavelength extending into the visible region of the electromagnetic spectrum (approximately 400 to 800 nm) occur in conjugated molecules [164]. Such stable colored radicals with powerful maximum absorption in the visible region diminish after an identified antioxidant or vitamin is included to determine the difference in absorption [161,162,163]. Energy absorbed by a molecule is spread over the molecule by some means such that absorption of radiation could force bonds to stretch or bend more energetically [164]. Also, absorption of radiation could result in an electron moving from a lower-energy orbital to a higher-energy orbital [164]. As a result, some antioxidants that provide free-radical inhibition measured chemically by spectrophotometer might not succeed at the cellular level or systemically on a physiological level [161]. Consequently, UV-Vis spectrophotometer measuring levels of energy changes surrounding molecular bonds does not correlate with vitamin clinical trials to prevent disease. On the other hand, covalent bonds formed during the free-radical polymerization tests by reactive secondary sequence for oils as unsaturated oleic and linoleic fatty acid lipids or vitamin A and beta-carotene that resulted in rubbery solid gels develop a basis for all consequential ROS pathology [1]. Pathological covalent bonds with macromolecular permanency are especially related to the basics of oxygen biochemistry for covalent bond sequestering of electrons and protons during mitochondrial energy synthesis to form water. Increased membrane fluidity is generally acknowledged to better facilitate diffusion for oxygen through cell membranes [165,166]. As oils becomes more viscous through ROS crosslinking with membrane fluidity lowered, membrane diffusion of O_2_ would also be reduced. As O_2_ transport is reduced through cell membranes with the subsequent possible interference of the electron transport chain in the mitochondria, production of ROS would be increased related to numerous diseases. When more ROS are generated, a vicious cycle of chronic increasing intermittent hypoxia with increased molecular crosslinks could develop that propagates different diseases in cells and through tissues.

## 14. Hydroquinone and Other Quinone Derivatives

**Figure 17:**
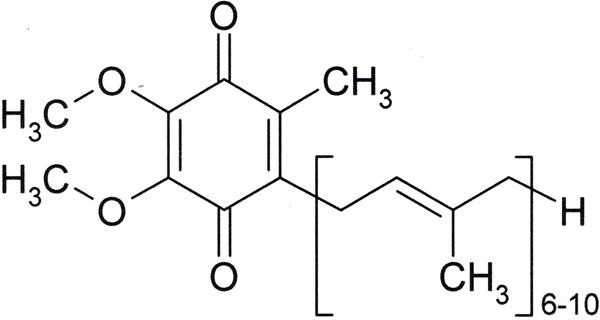
Ubiquinone or coenzyme Q10 electron transport molecule of the mitochondria.

The valuable antioxidant properties of fruits and vegetables may come from sources other than vitamins not yet recognized [145]. In fact, the most common antioxidants for ROS are identified as cellular proteins by the enzymes superoxide dismutases, catalase and glutathione peroxidase [27,79,80,86] with radicals delocalized into protein side chains and peptide bonds [87]. Also, coenzyme Q10 or ubiquinone, Figure 17, is a small molecular electron carrier of the inner mitochondrial membrane in the electron transport chain that has been recognized as an antioxidant and so used as a nonprescription dietary supplement [167]. Coenzyme Q10 as ubiquinone is a conjugate nonpolar quinone ring molecule found in the inner membrane of the mitochondria which both accepts an electron and also carries an acid through the lipid membrane barrier to create a proton gradient [75]. In 40 diabetes patients with significant impaired flow dilation of the brachial artery coenzyme Q10 improved endothelial performance with significant brachial artery dilation (*p* = 0.005) [168]. However, in retrospect molecules with similar structures to the coenzyme Q10 conjugated multiple double-bond side chain such as vitamin A and beta-carotene have increased mortality and cancer rates in some clinical trials [146,147,148].

**Figure 18:**
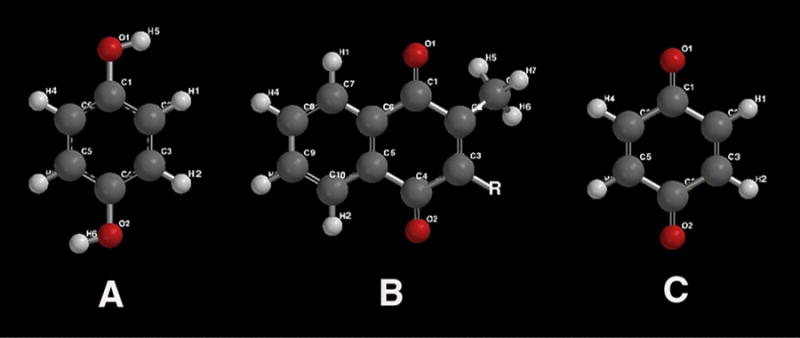
(A) Hydroquinone (B) Vitamin K (C) Quinone (Benzoquinone) (Micromechanics/Electron Interactions for Advanced Biomedical Research (2011) Chapter 16 Free Radical Reactive Secondary Sequence Lipid Chain-Lengthening Pathologies. Figure 17. Richard Petersen and Uday Vaidya).

Other quinones are used in dermatology, food preservatives, dietary supplements and as antioxidants to protect chemicals in polymer manufacturing. Hydroquinone is utilized as a reducing agent, antioxidant, free-radical inhibitor for polymerization, food preservative and an over-the-counter (OTC) nonprescription skin lightener to treat hyperpigmentation [169]. Structurally, the molecules hydroquinone and quinone or benzoquinone appear comparable to the quinone vitamin K, Figure 18, as conjugated planar ring molecules, but possibly more diffusive because of smaller molecular sizes. Quinone is the oxidized form of hydroquinone and will reduce back to hydroquinone [169,170]. The hydroquinone form of vitamin K has possible water solubility and demonstrates improved transport through biological fluids *in vivo* while the quinone vitamin K is almost insoluble in aqueous media [171].

Epidemiological studies in one hydroquinone manufacturing plant with 9040 workers representing 94,524 survival years over approximately a 10-year period found statistically significant decreases in mortality when comparing exposed workers to both non-exposed plant workers and the general population [169,172]. The same worker exposure study found statistically significant decreases in cancer rates, ischemic cardiovascular and cerebrovascular diseases, respiratory diseases and digestive diseases when comparing state and national vital statistics [169,172]. Another comprehensive epidemiology study of 858 men specifically exposed to hydroquinone representing 22,895 person-years at another plant for 48 years with an average exposure of 13.7 years revealed statistically significant decreases in mortality and cancer rates when comparing both non-exposed plant workers and the general population [168,173]. Other human studies at a manufacturing plant with significant levels of hydroquinone dust exposure showed no systemic toxicity [169,174].

Hydroquinone is well absorbed by oral ingestion [169]. No unfavorable hematological or urinary abnormalities occurred in two male volunteers with an oral intake of 500 mg/day for 5 months or in 17 male/female volunteers with 300 mg/day for 3 to 5 months [169,175]. For later comparison with animal toxicology testing, doses for a 70 kg person would be 7.14 mg/kg/day or 4.29 mg/kg/day respectively. Hydroquinone has been sold OTC since the 1950s and by prescription at different concentrations since 1961 [176,177,178] and is the most successful dermatology treatment for hyper-pigmentation with over 50 years of efficacy and safety data [177]. Further, hydroquinone use and manufacturing in over 50–60 years has resulted in no cases reported for cancer [177]. Hydroquinone is quickly and broadly absorbed after oral administration with subsequent rapid urinary removal with barely detectable residual remaining or bound to tissue [169]. Hydroquinone is excreted quickly by urinary elimination with humans following oral administration of a 4 mg/kg dose with a T_max_ for total hydroquinone in plasma of 1 hr and 90% removal by 5.6 hr [179]. Hydroquinone exposure also occurs to a considerable extent from natural plant dietary sources [169,179] with rapidly increased hydroquinone and metabolite plasma levels that peaked 5 times normal at 2 hours after eating hydroquinone-containing diets and much higher urinary excretion levels that peaked 12 times normal at 2–3 hours [179]. Recent fertility studies with modern bioassays using hydroquinone were unable to show reproduction toxicity [169,180]. Further, evidence is available suggesting that hydroquinone is protective in preventing hepatic carcinomas [169,180].

## 15. Polymerization Shrinkage Covalent Bond Antioxidant Tests for Vitamin E and Hydroquinone

**Figure 19:**
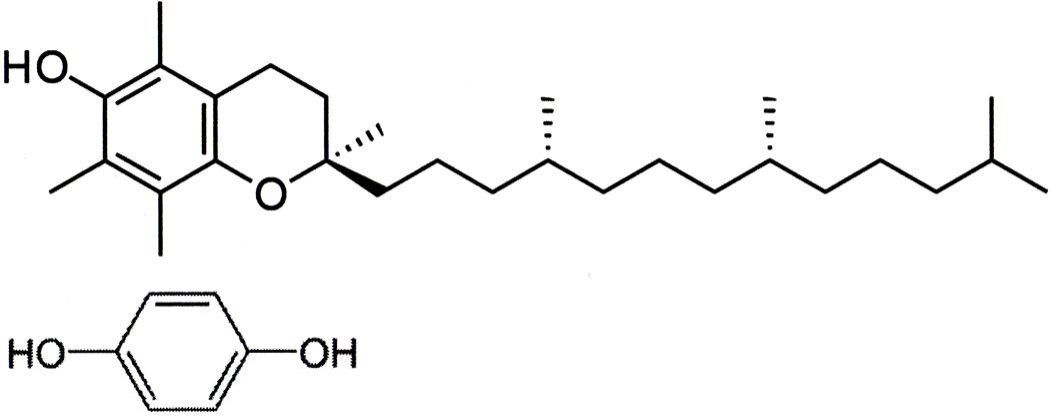
Vitamin E molecular structure compared to hydroquinone.

Vitamin E as the α-tocopherol form for antioxidant comparison with hydroquinone, Figure 19, has some molecular similarity to hydroquinone with an aromatic hydroxyl group to act as an antioxidant. However, the vitamin E aromatic ring is fully substituted with molecular groups whereas hydroquinone has four unsubstituted aromatic positions that can be effectively activated by two hydroxyl groups for reactivity with a strong electrophile like a free radical [170]. Thus, electrophilic aromatic substitution reactions with hydroquinone or p-dihydroxybenzene appear to occur as the chief antioxidant mechanism to scavenge free-radical electrophiles. Also, α-tocopherol is a much larger hydrophobic or nonpolar molecule than hydroquinone and from laboratory observations is practically insoluble in water whereas hydroquinone will dissolve easily to diffuse through water.

To evaluate the covalent-bond reaction for antioxidant performance between vitamin E and free-radical inhibitor hydroquinone, controls as unsaturated lipid:acrolein models at 46:46 wt% each were mixed with Fenton redox couples benzoyl peroxide initiator 4 wt% and cation transition metal cobalt naphthenate accelerator 4wt% to produce free radicals [1]. For comparisons, identical control groups were combined with different amounts of either vitamin E ((±)-α-tocopherol) or hydroquinone [1]. Shrinkage was thus calculated over time for 50 hours by measuring the differences between the original level for the volume and volumetric shrinkage polymerization level as a relative measure of covalent bond crosslinking.

**Figure 20:**
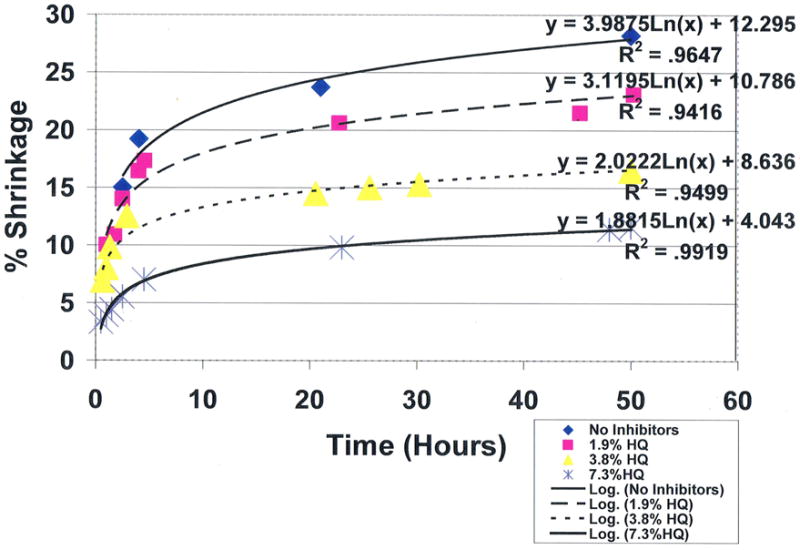
Lipid and acrolein free-radical covalent bonding polymerization shrinkage with hydroquinone free-radical inhibitor at different concentrations. (International Research Journal of Pure &Applied Chemistry 2(4): 247–285, (2012) Reactive Secondary Sequence Oxidative Pathology Polymer Model and Antioxidant Tests. Figure 15. Richard Petersen).

**Figure 21:**
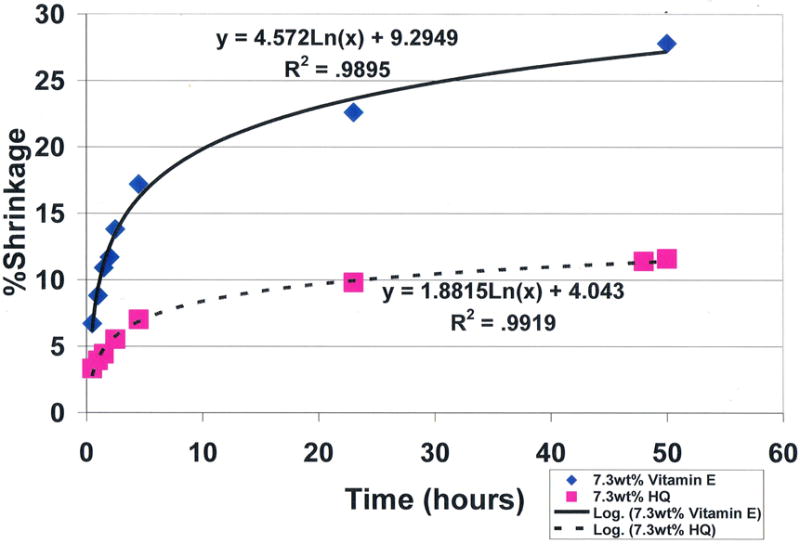
Lipid and acrolein free-radical covalent bonding polymerization shrinkage comparing antioxidant free-radical sequestering with 7.3 wt% hydroquinone and 7.3 wt% vitamin E. (*p* < 0.00001 at 50 hrs) (International Research Journal of Pure &Applied Chemistry 2(4): 247–285, (2012) Reactive Secondary Sequence Oxidative Pathology Polymer Model and Antioxidant Tests. Figure 16. Richard Petersen).

Results for hydroquinone showed impressive statistically significant concentration dependent antioxidant properties for removing free radicals with reductions in polymerization shrinkage during 50-hour test periods from the 28.2% control baseline at 0.0 wt% down to 11.6% at 7.3 wt% (*p* < 0.0001), Figure 20. Antioxidant testing showed a dominating statistical significant improvement in free-radical inhibition for 7.3 wt% hydroquinone over 7.3 wt% vitamin E that demonstrated no antioxidant activity for scavenging free radicals with polymerization shrinkage of 27.8% after 50 hours, (*p* < 0.00001). Hydroquinone and vitamin E are compared simultaneously at 7.3 wt% each in Figure 21. Of interest to outer-shell electron transfer during free-radical covalent bonding with all reactants and hydroquinone scavenging of free radicals, all reaction rates in terms of overall products formed were logarithmic rather than linear as predicted by the Marcus Theory [181]. In terms of the amount for variation or percent explained from the natural log equations obtaining R^2^ values ranging from 0.9416 to 0.9919 is then 94.16% to 99.19%.

Results have clearly demonstrated that vitamin A and beta-carotene nutrient oil supplements can crosslink into solids when exposed to a sufficiently high concentration of free radicals while vitamin E has no antioxidant inhibitory effects to prevent free-radical crosslinking of unsaturated lipids common to cell membranes [1]. Free radicals have been shown to reduce membrane fluidity [26,31–35] most generally seen with PUFAs that are decreased as an indication crosslinking occurs [31–35]. Subsequent reduction in membrane fluidity reduces molecular oxygen diffusion through cell membranes [165,166,182]. Free-radical crosslinking in cell membranes would then result in molecular obstructions that compromise oxygen transport to reduce diffusion and create possible ROS build-up from the mitochondrial electron transport chain during energy synthesis. On the other hand, hydroquinone proved highly valuable in reducing free-radical induced covalent bond crosslinking in an unsaturated lipid solution with a strong crosslinking lipid breakdown aldehyde product acrolein [1]. The fact that vitamin A, beta-carotene and vitamin E are the most studied nutrients for antioxidants in diets that reduce risks in cancer and cardiovascular incident but have failed in clinical trials as vitamin supplements has raised the question that other antioxidant molecules not yet identified may be available for treatment [145]. For a chief antioxidant source, hydroquinone is found in plants as the glucose conjugate 4-hydroxyphenyl-β-D-glucopyranoside or arbutin [179]. Arbutin hydrolyzes without difficulty in low acid concentrations to give glucose and hydroquinone so that arbutin would release hydroquinone in the digestive process [169,179]. Further, considerable hydroquinone has been shown to occur in the urine and plasma from plant dietary sources [169,179]. ROS have been recognized as key reasons for many diseases including cancer and cardiovascular disease to include aging. Therefore, because hydroquinone and many derivatives of hydroquinone are well-known free-radical inhibitors, the possibility exists that new and real antioxidant treatment options may be available for numerous diseases.

## 16. Hydroquinone OTC Use

Despite long-term efficacy and safety data with hydroquinone and human epidemiology studies showing a reduction in cancer and mortality by hydroquinone spanning up to 50–60 years, the Food and Drug Administration (FDA) with an unusual decision in 2006 proposed to withdraw OTC use of hydroquinone skin-lightening products. Levitt in response to the FDA recommendation provides clear informed details showing the proposal is unreasonable [180]. The FDA made the recommendation to remove hydroquinone OTC for human use mainly because of outdated rodent studies in 1989 [183] and 1991 [184] indicating some evidence shows hydroquinone may act as a carcinogen after heavy chronic 2-year parental gavage administration or oral daily diet. However, doses in the 2-year F-344 rat studies included a five days per week dose by parental gavage that ranged from 25–50 mg/kg [183] or daily oral intake at 351 mg/kg for male rats and 368 mg/kg for female rats [184]. On the other hand, by comparison much lower experimental daily oral doses were delivered for an approximate 70 kg human weight at 7.14 mg/kg for 5 months with the two males or 4.29 mg/kg for 3–5 months with 17 males/females [169,175]. So, in terms of error for comparisons oral doses for hydroquinone with the much longer rat toxicology study were on the order of about 50 to 80 times higher than the shorter-term human hydroquinone experiment. Regarding safety, comparison with a well-designed randomized ATBC clinical trial of 29,133 male Finnish smokers taking daily nutrient β-carotene supplements at a much lower oral dose of just 20 mg or 0.3 mg/kg for a 70 kg person during an average of 6.1 years statistically significantly increased risk of lung cancer 18% and overall mortality 8% [146]. The daily oral dose for the β-carotene ATBC human study that resulted with increased cancer risk and mortality was over a 1000 times lower [146] than the oral dose for the hydroquinone F-344 rat study [184]. The CARET randomized clinical trial with a combination of β-carotene and vitamin A for an oral dose of 30 mg daily at about 0.4 mg/kg for a 70 kg male resulted in a 28% increase in cancer and 17% increase in total mortality [148]. The CARET nutrient supplement clinical trial would then deliver a dose on the order of almost 1000 times lower than the oral hydroquinone rat F-344 study.

Of particular importance, hydroquinone toxicity in animals is generally restricted to chronic progressive nephropathy (CPN) in male F-344 rats that further showed signs of increased renal adenomas [169,176,180]. However, humans do not develop CPN [169] and *in vivo* topical or oral exposure to hydroquinone has never shown any signs of toxicity on human kidney function [169,176,177]. Metabolism of hydroquinone following oral ingestion into reactive conjugates appears to be a strong factor in renal toxicity and CPN leading to renal tumors in laboratory animals [169]. But, human hepatocytes detoxify hydroquinone conjugates at a greater capacity than rats [185]. No increase in mortality was recognized as a result of renal disease or tumors in a human mortality study at a large hydroquinone manufacturing facility [173]. Renal cancer is sex, species, strain and age specific and shows no significance to use of hydroquinone in humans after multiple decades with extensive use of hydroquinone [180]. In terms of mutagenicity, hydroquinone has not been shown to be a carcinogen by Ames test [177]. In over 50–60 years hydroquinone has been used without any related cancers reported [177]. All regulatory divisions previously determined there were not sufficient facts to classify hydroquinone as a carcinogen [177] prior to the unusual FDA OTC recommendation based on old rodent carcinogenic tests [183,184]. Because species other than F-344 rats do not form renal adenomas from hydroquinone, and no renal effects from hydroquinone are observed in humans, and negligible mutagenic probability exists for hydroquinone, nephrotoxicity or renal cancer is not a pertinent risk in humans [169]. Expert evaluation of extensive rodent carcinogenicity studies has created an understandable awareness that chronic rodent tests over-predict risks for humans [186]. Nonetheless, ignoring all epidemiological and safety references on human hydroquinone exposures and obvious problems with gender and species nephrotoxicity with hydroquinone, tests for human relevance on hydroquinone were proposed in 2009 to study metabolism and reproductive toxicity by oral and dermal routes and dermal carcinogenicity studies in both rats and mice. However, still much later in a continuing unique response as of November 29, 2015 before the rodent studies begin the FDA recommends that hydroquinone should still remain available as an OTC drug product for treating hyper-pigmentation [187].

## 17. Conclusions

Membrane fluidity is important to provide translational motion for lipids and protein. The fluidity of a membrane can be decreased with free radicals by crosslinking through PUFAs. Subsequent loss of membrane fluidity reduces oxygen diffusion. Lower oxygen diffusion in cell membranes ultimately lowers oxygen availability for mitochondria during energy synthesis through a form of intermittent hypoxia to generate more free radicals. Thus, a spiraling effect of higher free-radical concentrations with increased membrane PUFA crosslinking for greater membrane rigidity and larger macromolecular barriers reduces membrane fluidity even more. Lowering membrane fluidity then further decreases oxygen membrane diffusion in an increasing vicious cycle for mitochondria to produce more free radicals that can damage lipids, proteins and DNA. Accumulation of free radicals and free-radical damage is thought to be a chief reason for aging, age-related diseases, and promote most medical conditions. Epidemiological studies have identified fruits and vegetables as sources of potential free-radical scavenging antioxidants that limit many diseases to include cancer and cardiovascular disease. However, unreliable colorimeter antioxidant testing has erroneously identified many nutrient supplements such as beta-carotene and vitamins A, C and E that have failed in clinical trials and even increased diseases with increased mortality in some studies. On the other hand, hydroquinone is an exceptionally efficient free-radical inhibitor designed to sequester free radicals and also found in dietary sources. Further, hydroquinone was tested accurately by covalent bond free-radical polymerization shrinkage measurements with statistically significantly improved antioxidant results over vitamin E, *p* < 0.00001. Hydroquinone is a safe and effective OTC dermatological drug for treatment of hyper-pigmentation and has shown in large long-term worker exposure epidemiology studies to significantly statistically reduce cancer and many other major diseases and also significantly statistically reduce total mortality. Consequently, hydroquinone is a new drug therapy possibility as a true antioxidant and free-radical inhibitor for many potential disease states.

## Figures and Tables

**Table 1:** Chemical formulas of different potential calcium and magnesium compounds [47].

Compound	Abbreviation	Formula	Ca/P
Dicalcium phosphate	DCP	CaHPO_4_	1.0
Dicalcium phosphate dehydrate	DCPD	CaHPO_4_.2H_2_O	1.0
Micellar calcium phosphate	MCP	Ca(HPO_4_)_0.7_(PO_4_)_0.2_· × H_2_O	1.1
Octacalcium phosphate	OCP	Ca_8_H_2_(PO_4_)_6_·5H_2_ O	1.33
β-Tricalcium phosphate	β-TCP	β-Ca_3_(PO_4_)_2_	1.5
Hydroxyapatite	HAP	Ca_5_OH(PO_4_)_3_	1.67
Amorphous calcium phosphate	ACP	Ca_3_(HPO_4_)_0.2_(PO_4_)_1.87_ × H_2_ O	1.45
Tricalcium citrate dehydrate	TCC	Ca_3_(Cit)_2_·2H_2_O	–
Dimagnesium phosphate	–	MgHPO_4_	–

**Table 2:** Resistivity^a^ of Material Relationships to Calcium and Membrane Phosphate Headgroups.

Material	Type	Resistivity (Ωm)
Gold (Best Conductor)	Conductor	2.21 × 10^−8^ [63]
Calcium	Conductor	3.36 × 10^−8^ [63]
Magnesium	Conductor	4.39 × 10^−8^ [63]
General Metals	Conductors	~10^−6^–10^−8^ [66]
Physiologic Saline	Semiconductor	0.7 [67]
Bisphenyl Matrix Carbon Fiber Composite	Semiconductor	5 [65]
Lipid Phosphate Headgroup/Water Interface	Semiconductor	100 [64]
Silicon Pure	Semiconductor	3000 [68]
General Polymers	Insulators	~10^8^–5 × 10^16^ [66]
Sulfur	Insulator	2 × 10^15^ [69]
Polyethylene (HDPE and LDPE)	Insulator	10^15^–5 × 10^16^ [66]

a Resistivity = 1/Conductivity
